# Supplementary material for: Enantioselective Total Synthesis of Daedaleanol B from (+)-Sclareolide
Source: Molecules. 2026 Jan 4;31(1):185. doi: 10.3390/molecules31010185 (PMC12787746; doi:10.3390/molecules31010185)
Supplement: Supplementary file 1 [file molecules-31-00185-s001.zip › molecules-4083301-supplementary.pdf]

Supporting Information

Enantioselective total synthesis of daedaleanol B from (+)-sclareolide

Irene Moreno-Gutiérrez,[1] Sonia Berenguel-Gómez,[1] María José Cánovas-Aragón,[1] José Luis Guil-Guerrero,[2] Tarik Chileh-Chelh,[2] Manuel Muñoz-Dorado,[1] Miriam Álvarez-Corral[1*] and Ignacio Rodríguez-García[1]*

1 Organic Chemistry, CeiA3, CIAIMBITAL, University of Almería. 04120 Almería, Spain; img823@ual.es (I. M.-G.); sbg479@ual.es (S.B..-G.); mdorado@ual.es (M.M.-D.); malvarez@ual.es (M.A.-C.); irodrigu@ual.es (I.R.-G.)

2 Food Technology Division, ceiA3, CIAMBITAL, University of Almería. 04120 Almería, Spain; jlguil@ual.es (J.L. G.-G.); chileh@hotmail.es (T.C.-C.)

* Correspondence: malvarez@ual.es (M.A.-C.); irodrigu@ual.es (I.R.-G.)

NMR, IR AND CD SPECTRA. X-RAY DIFFRACTION DATA. HPLC CHROMATOGRAM.

^{1}H NMR, DEPT 135, ^{13}C NMR and IR of (*E,E*)-farnesyl acetate....................1
^{1}H NMR, DEPT 135, ^{13}C NMR, 1D-NOE and IR of **11**....................4
^{1}H NMR of **9** and **12** mixture....................8
^{1}H NMR of **15**....................9
^{1}H NMR of **16**....................10
^{1}H NMR of **17**....................11
^{1}H NMR, DEPT 135, ^{13}C NMR, COSY, HSQC, HMBC and IR of **6**....................12
^{1}H NMR, DEPT 135, 13C NMR, circular dichroism, X-Ray diffraction and IR of **13**....................18
^{1}H NMR, DEPT 135, ^{13}C NMR, COSY, HMBC, HSQC and NOESY of **14**....................23
^{1}H NMR, DEPT 135, ^{13}C NMR, HSQC, COSY and IR of **5**....................29
HPLC chromatogram for the separation of **5** and **14**....................35

^{1}H NMR, DEPT 135, ^{13}C NMR and IR of (*E*,*E*)-farnesyl acetate

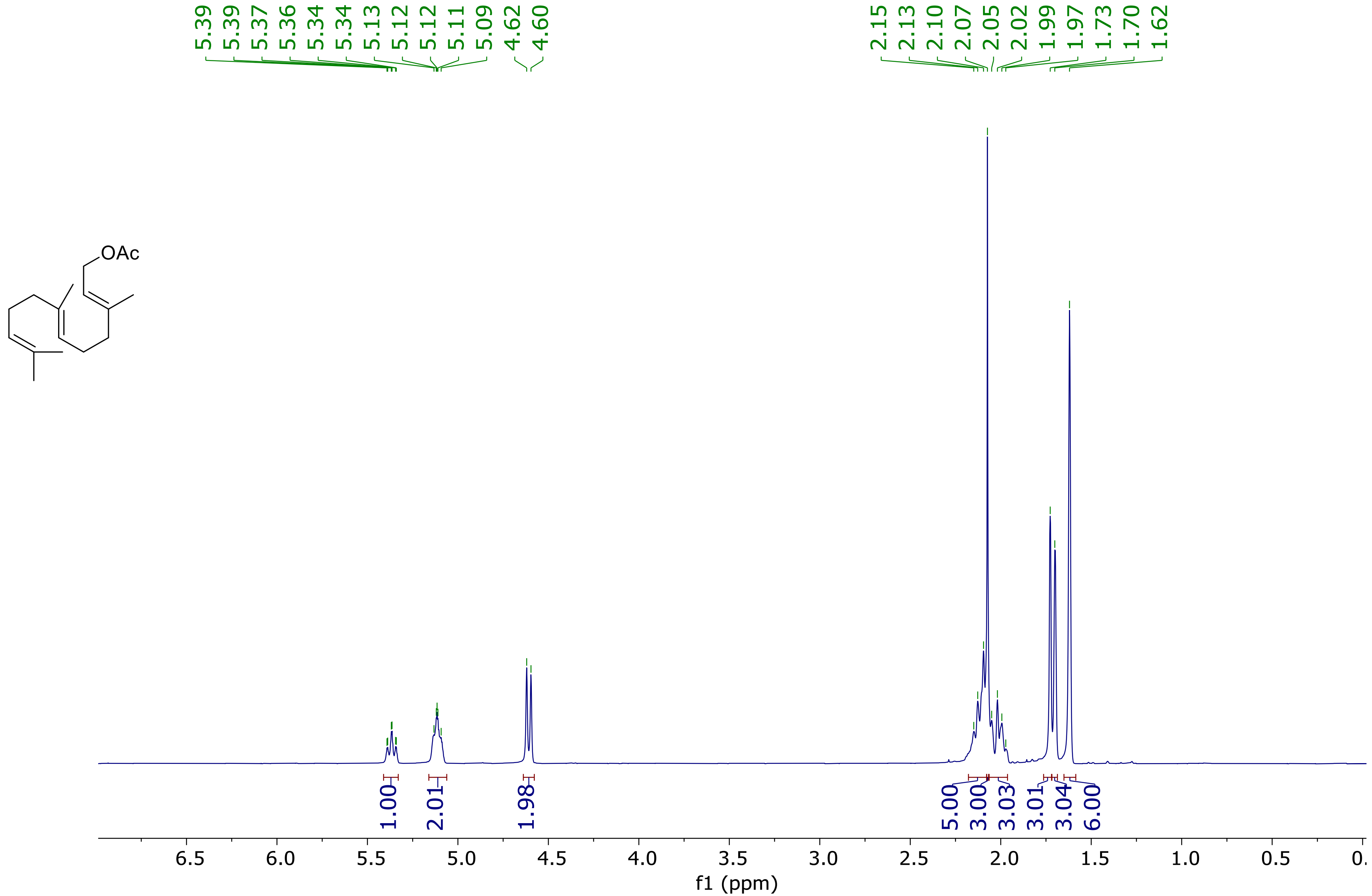

Figure S1. ^{1}H NMR (300 MHz, $CDCl_3$) of (*E*,*E*)-farnesyl acetate.

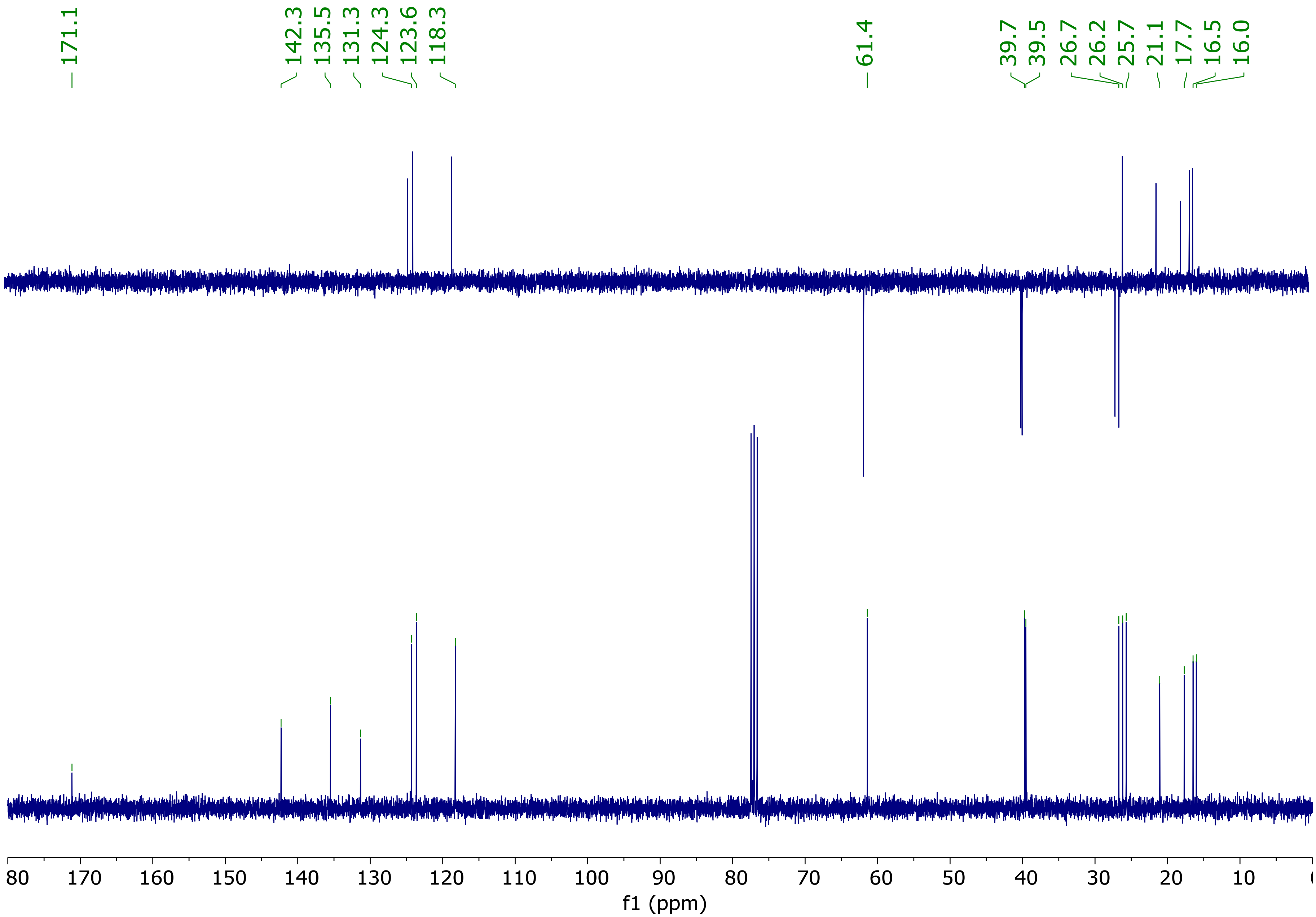

Figure S2. ¹³C NMR and DEPT (75 MHz, $CDCl_3$) of (*E*,*E*)-farnesyl acetate.

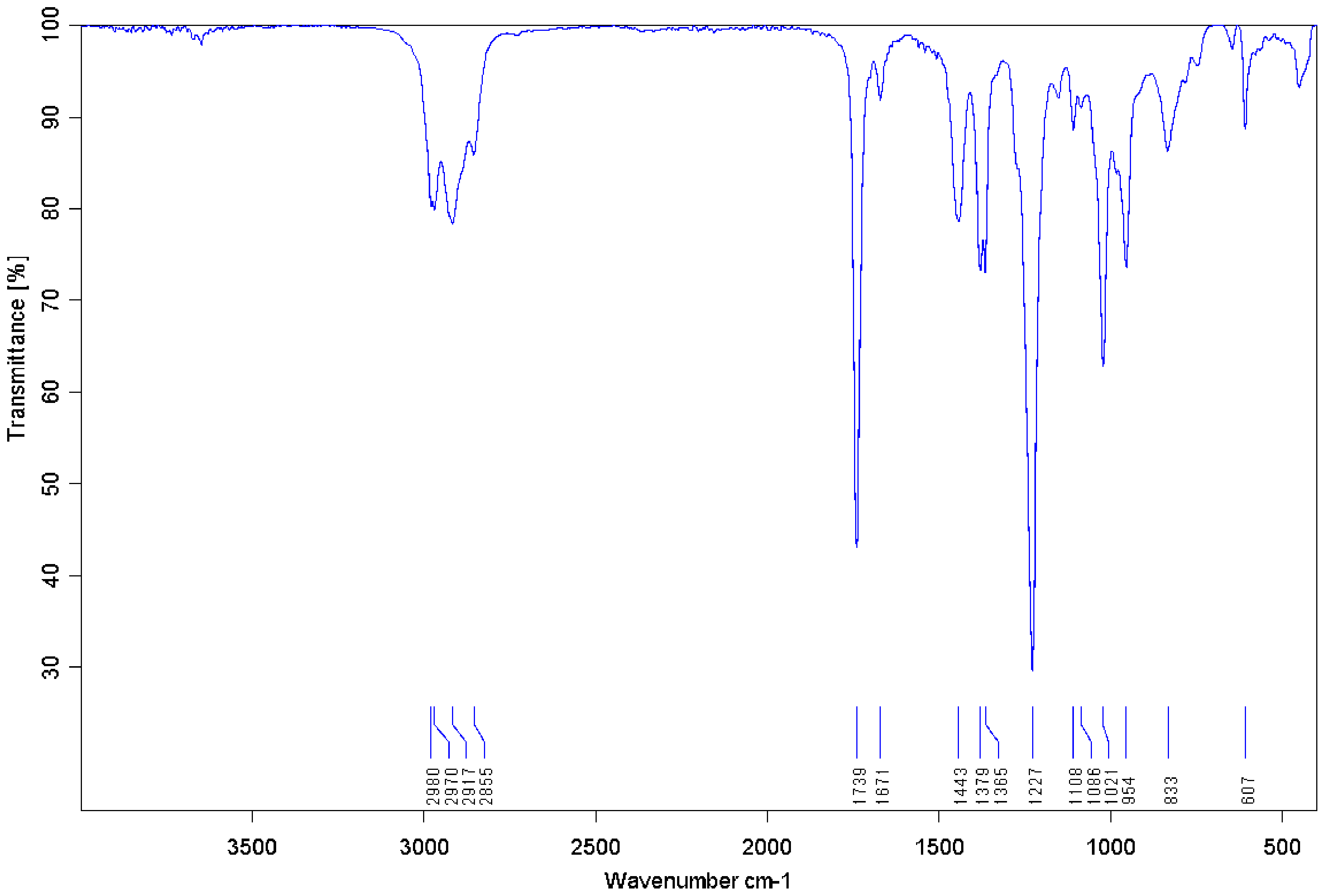

Figure S3. IR (ATR) of (*E,E*)-farnesyl acetate.

^{1}H NMR, DEPT 135, ^{13}C NMR, 1D-NOE and IR of 11

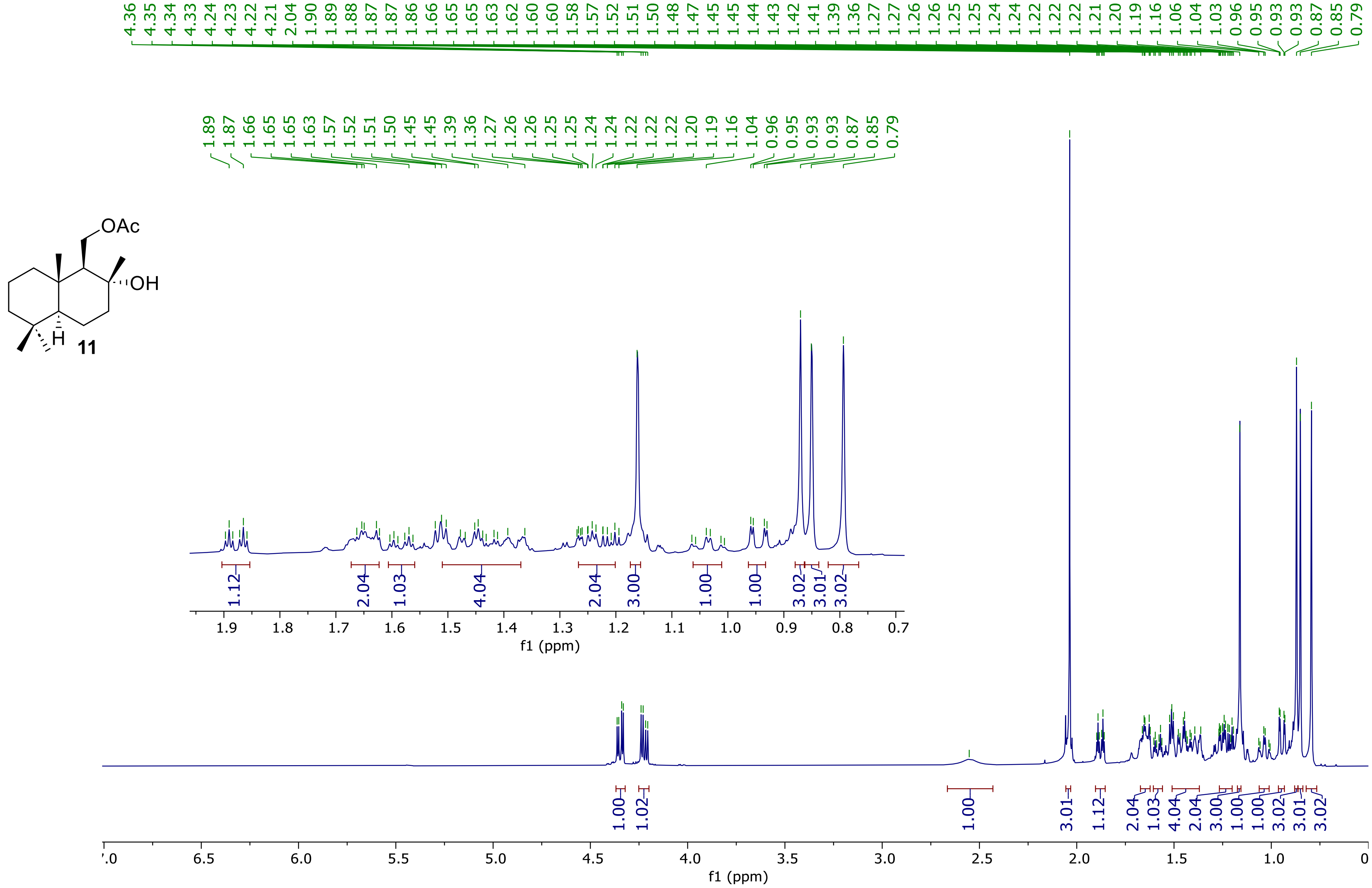

Figure S4. ^{1}H NMR (500 MHz, $CDCl_3$) of **11**.

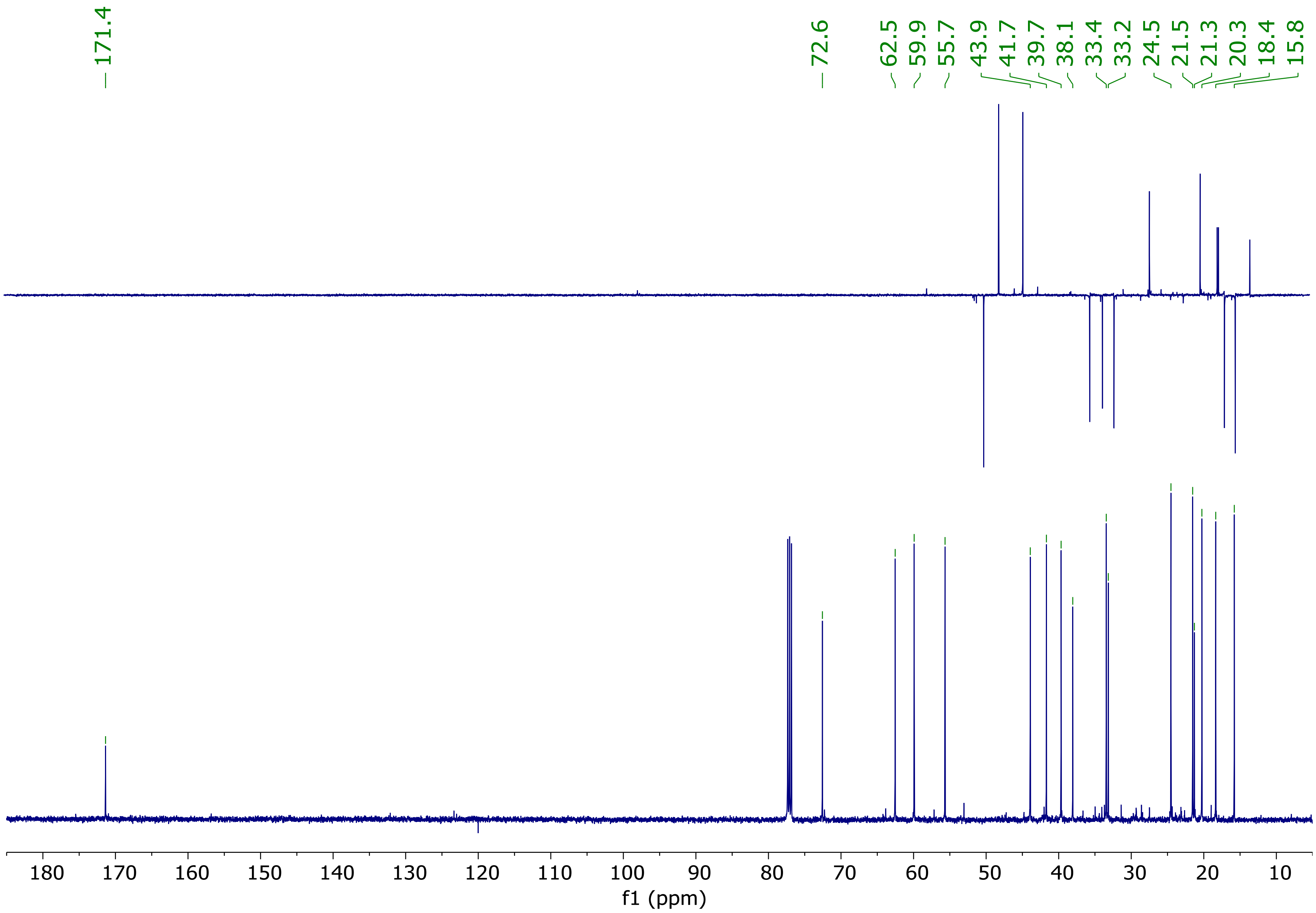

Figure S5. ^{13}C NMR (125 MHz, $CDCl_3$) of **11**.

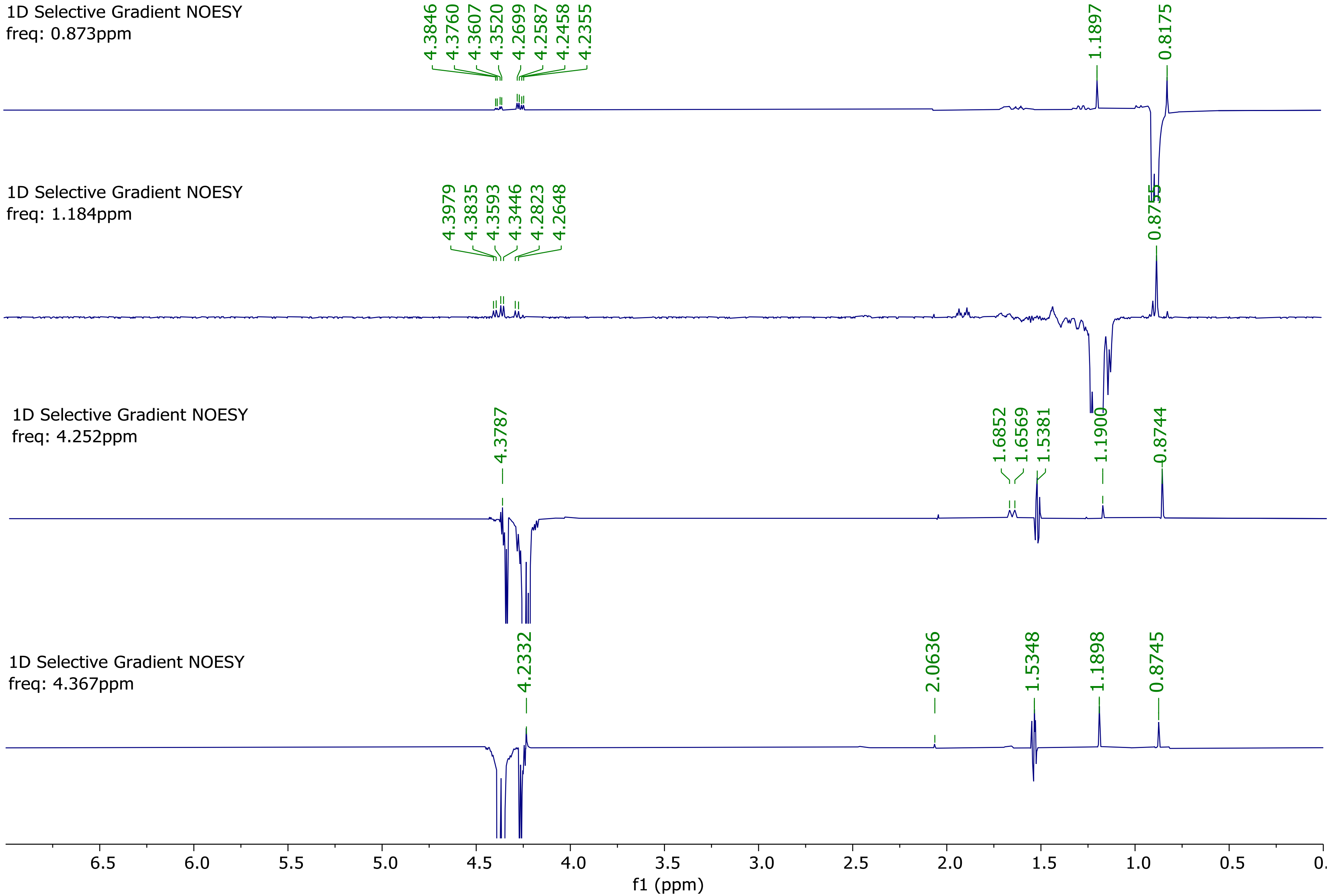

Figure S6. ^{1}H NMR 1D selective gradient NOESY (300 MHz, $CDCl_3$) of **11**.

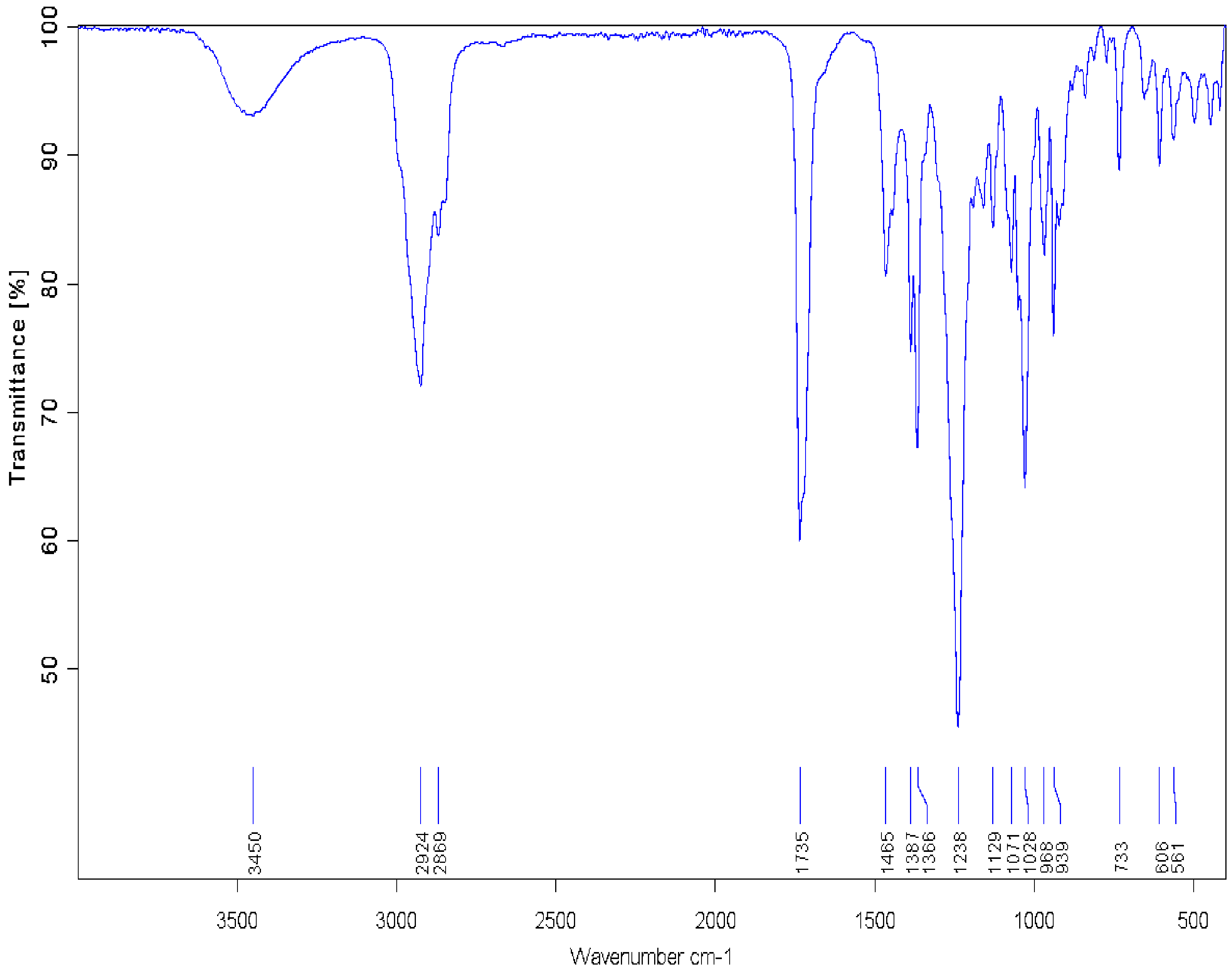

Figure S7. IR (ATR) of **11**.

^{1}H NMR of 9 and 12 mixture

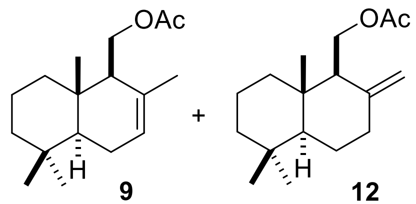

9 12

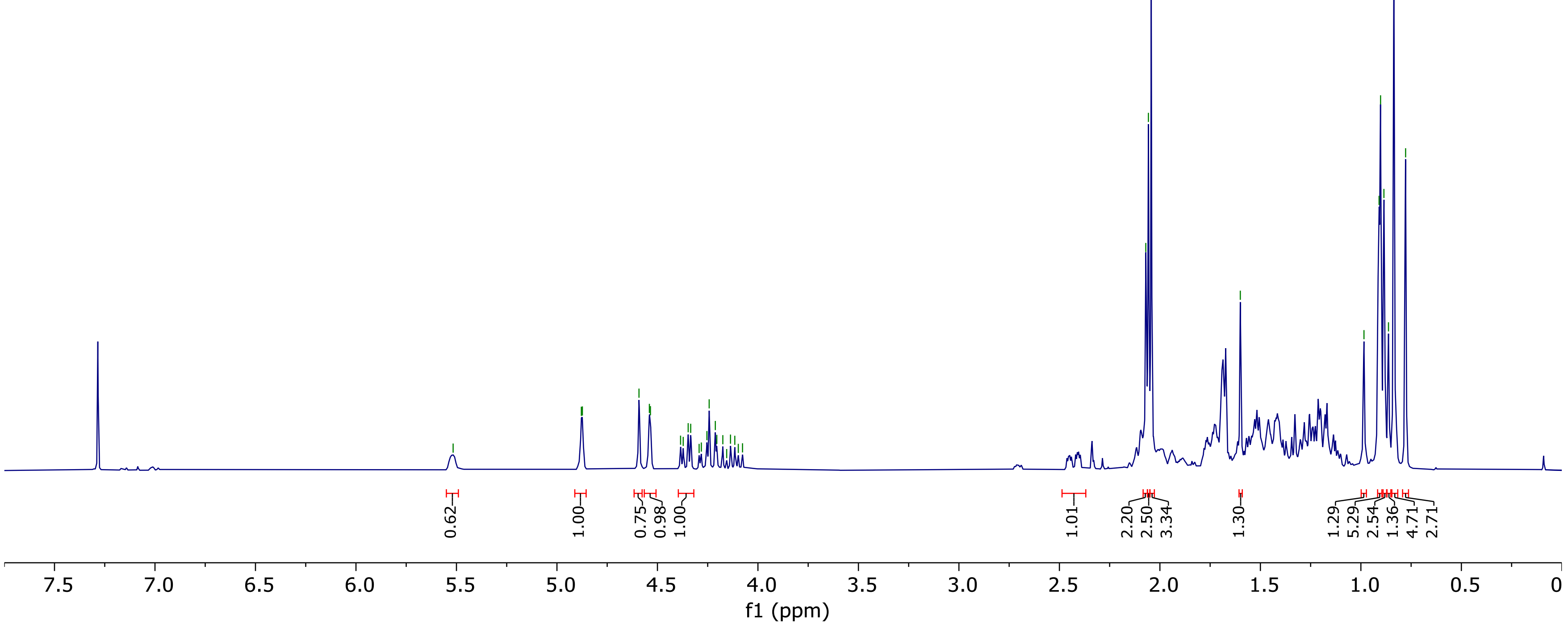

Figure S8. ^{1}H NMR (300 MHz, $CDCl_3$) of the crude reaction mixture of **9** and **12**.

^{1}H NMR of 15

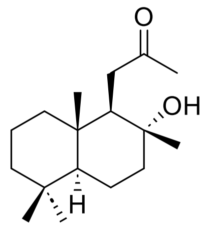

15

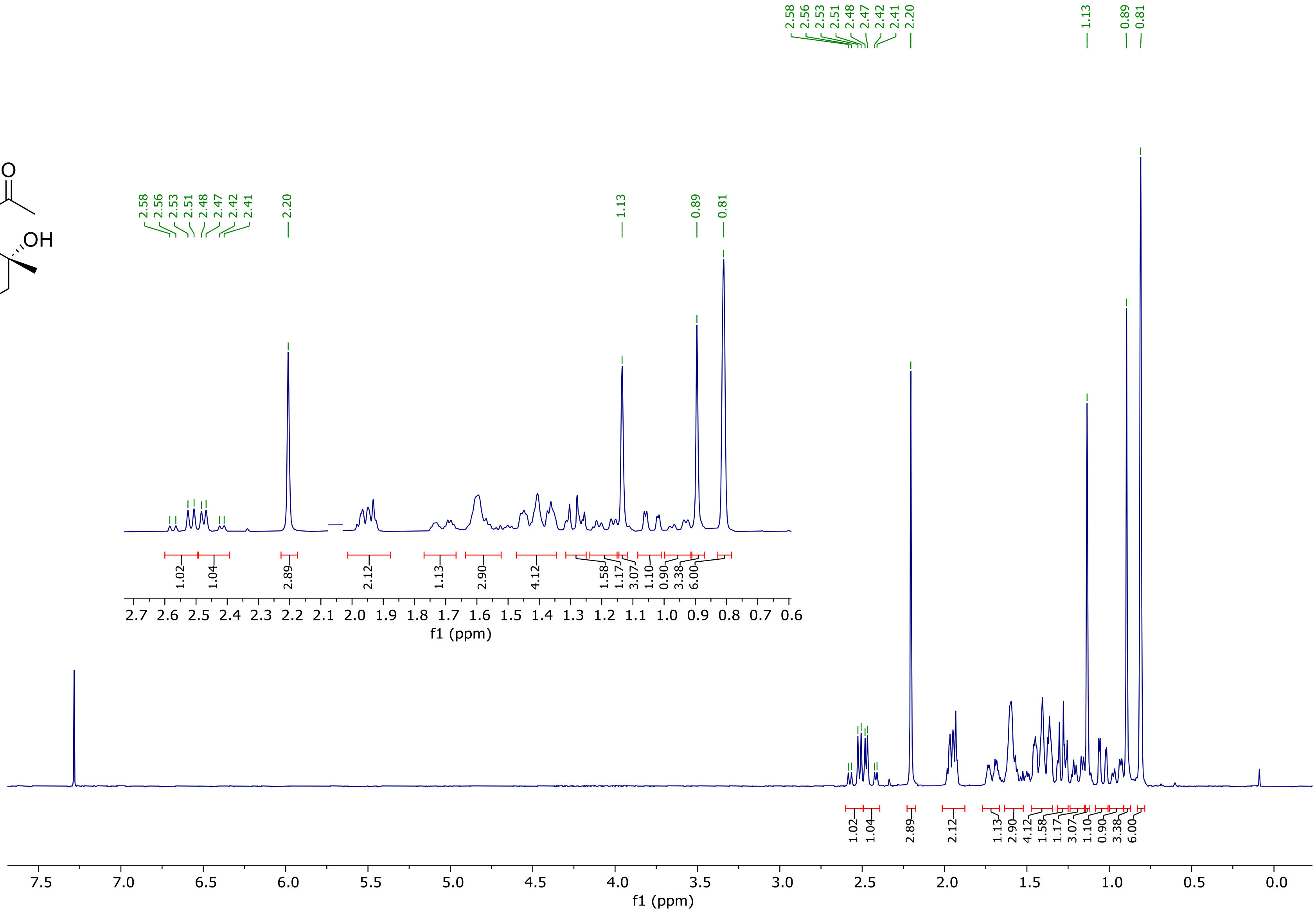

Figure S9. ^{1}H NMR (300 MHz, $CDCl_3$) of **15**.

^{1}H NMR of 16

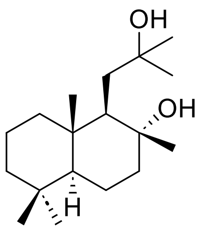

16

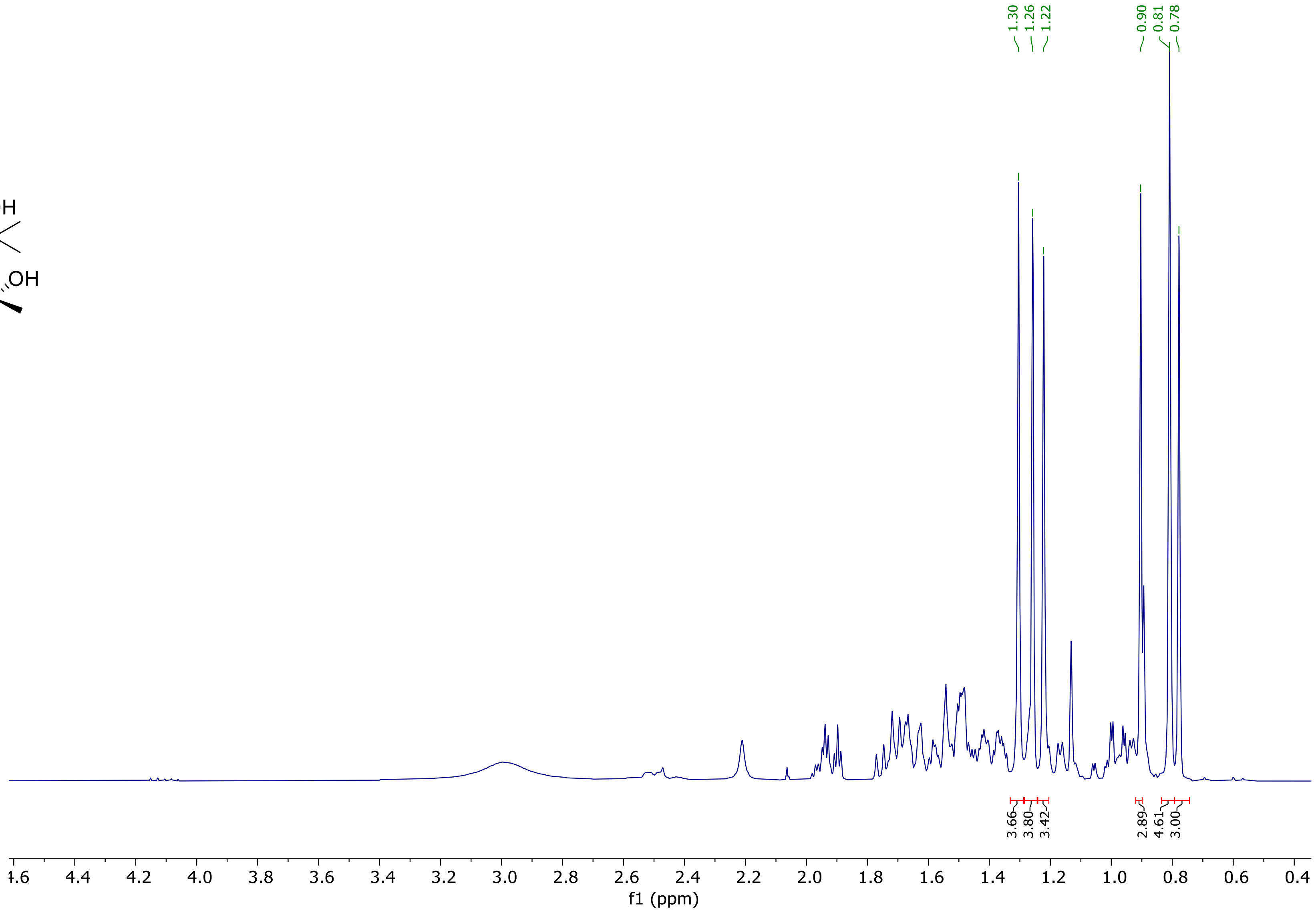

Figure S10. ^{1}H NMR (300 MHz, $CDCl_3$) of **16**.

^{1}H NMR of 17

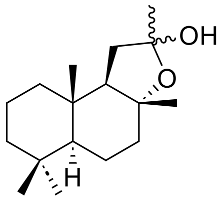

17

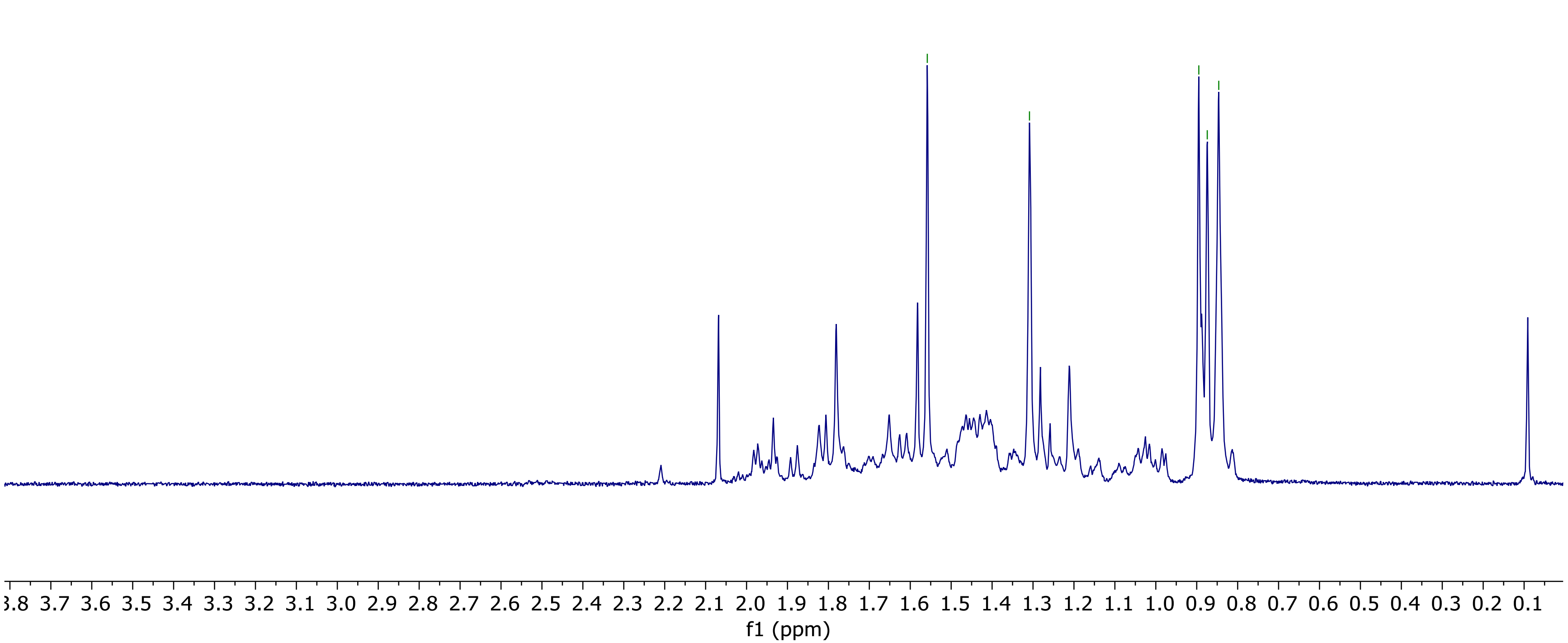

Figure S11. ^{1}H NMR (300 MHz, $CDCl_3$) of **17**.

^{1}H NMR, DEPT 135, ^{13}C NMR, COSY, HSQC, HMBC and IR of 6

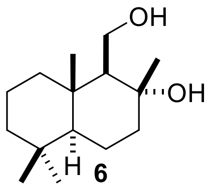

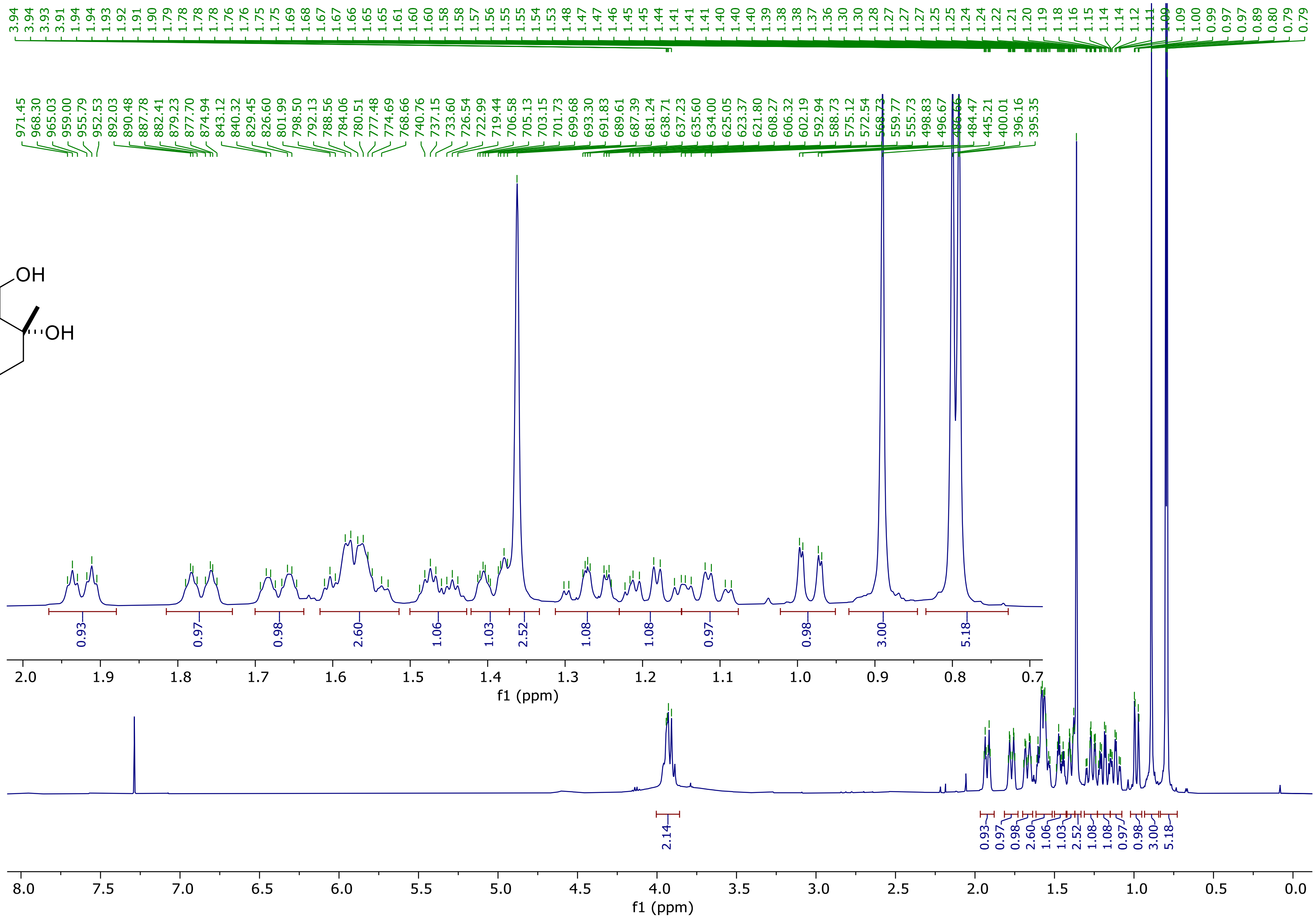

Figure S12. ^{1}H NMR (500 MHz, $CDCl_3$) of **6**.

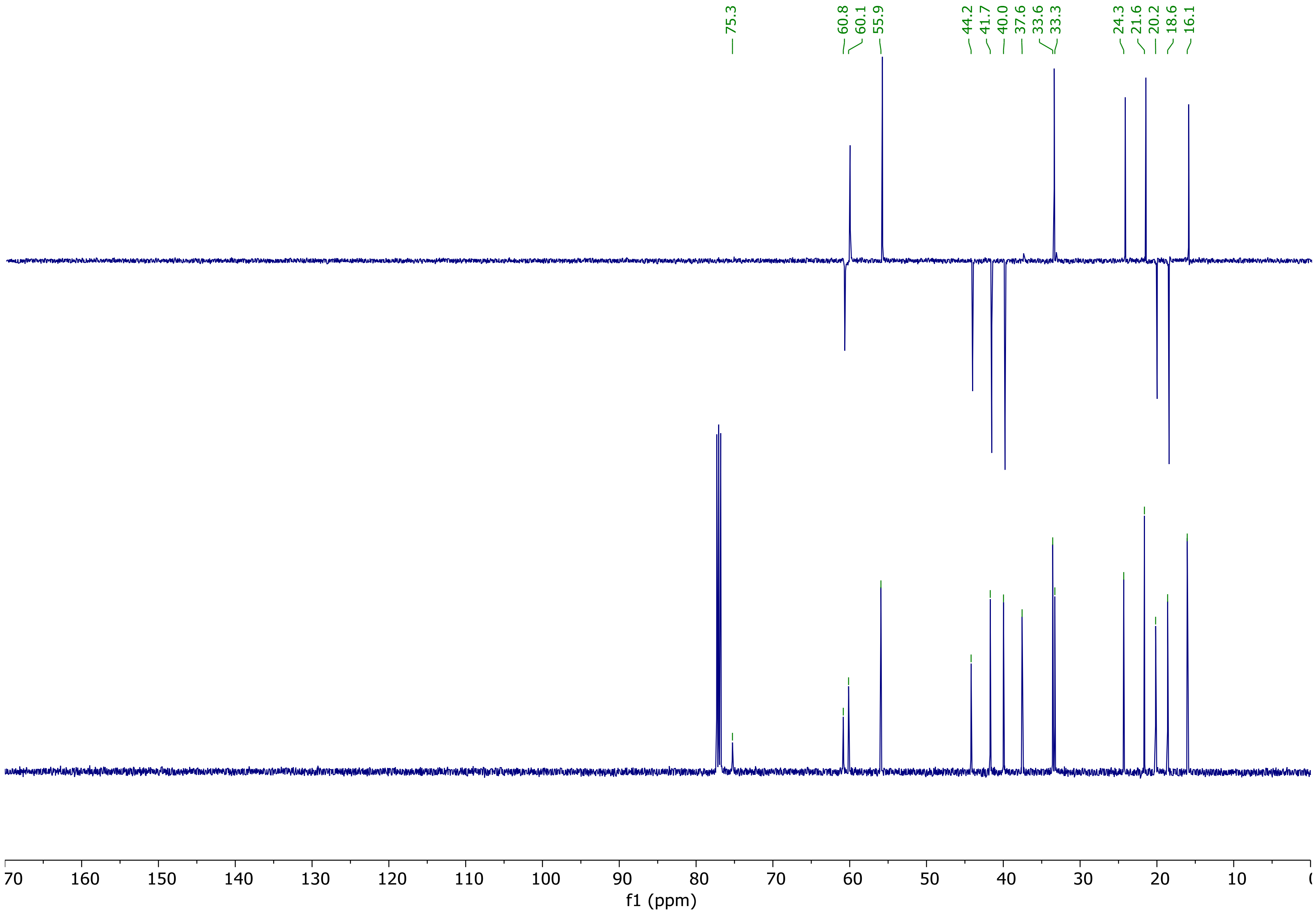

Figure S13. ^{13}C NMR and DEPT (125 MHz, $CDCl_3$) of **6**.

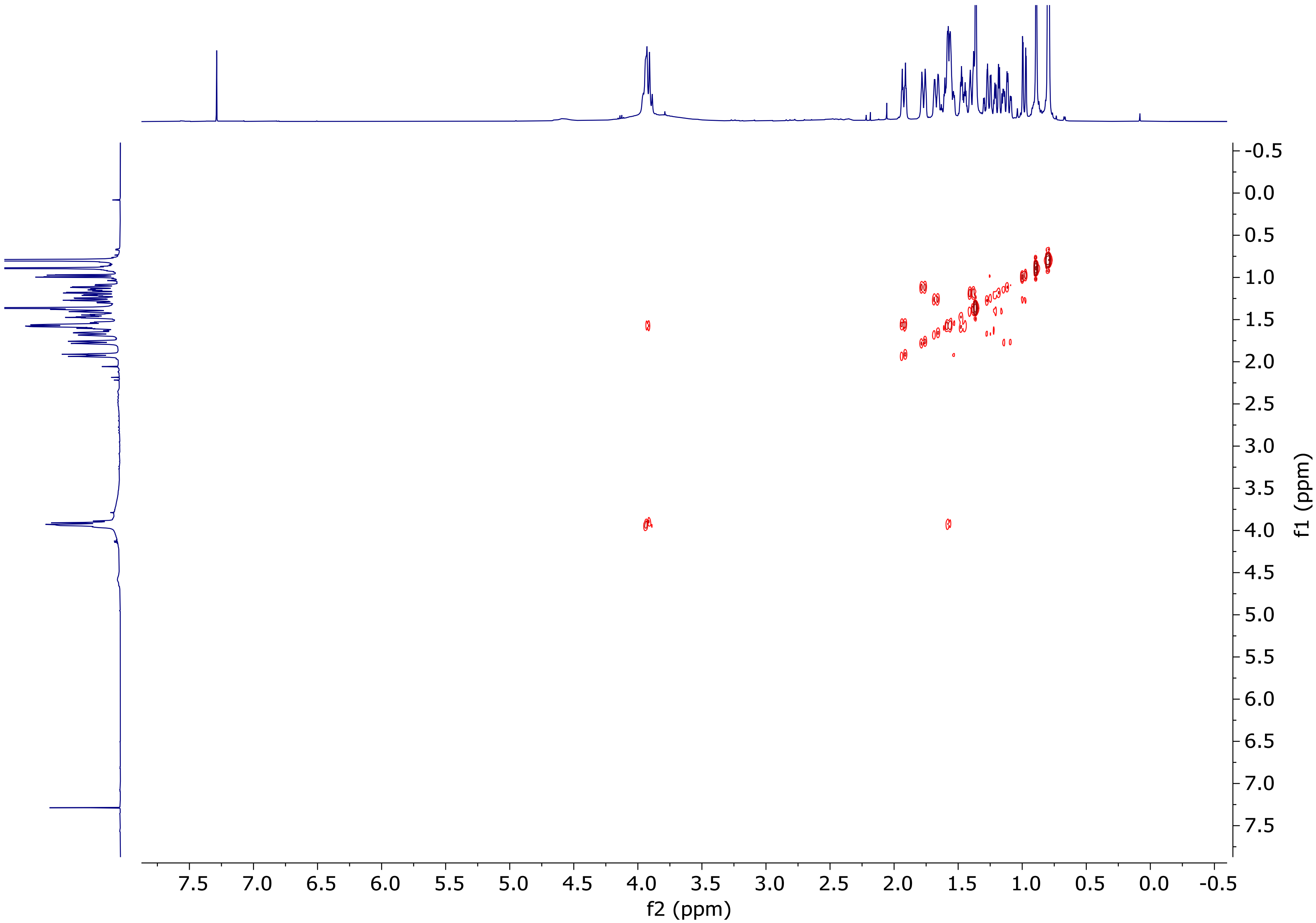

Figure S14. 2D NMR (COSY) (500 MHz, $CDCl_3$) of **6**.

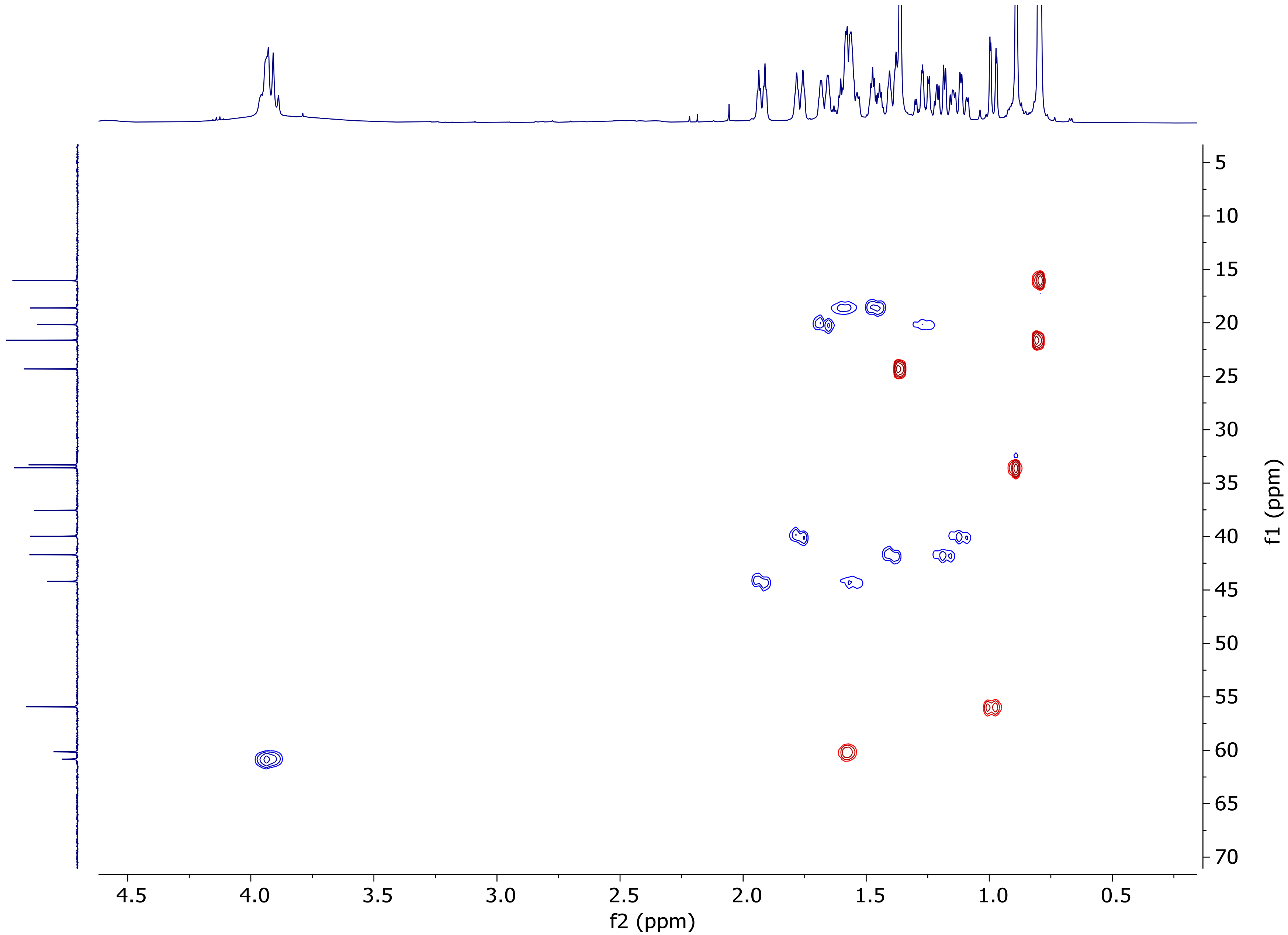

Figure S15. 2D NMR (HSQC) (500 MHz, $CDCl_3$) of **6**.

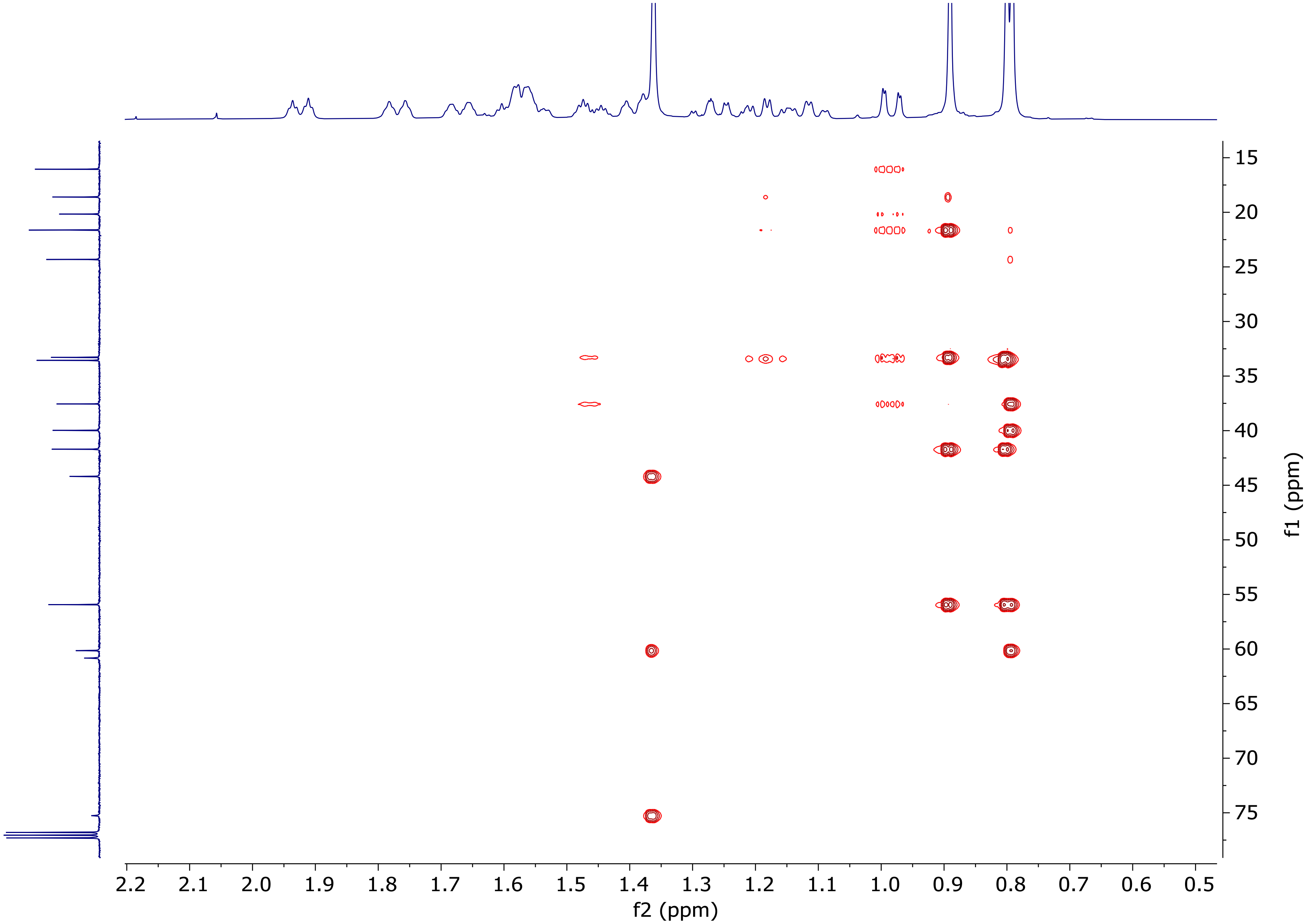

Figure S16. 2D NMR (HMBC) (500 MHz, $CDCl_3$) of **6**.

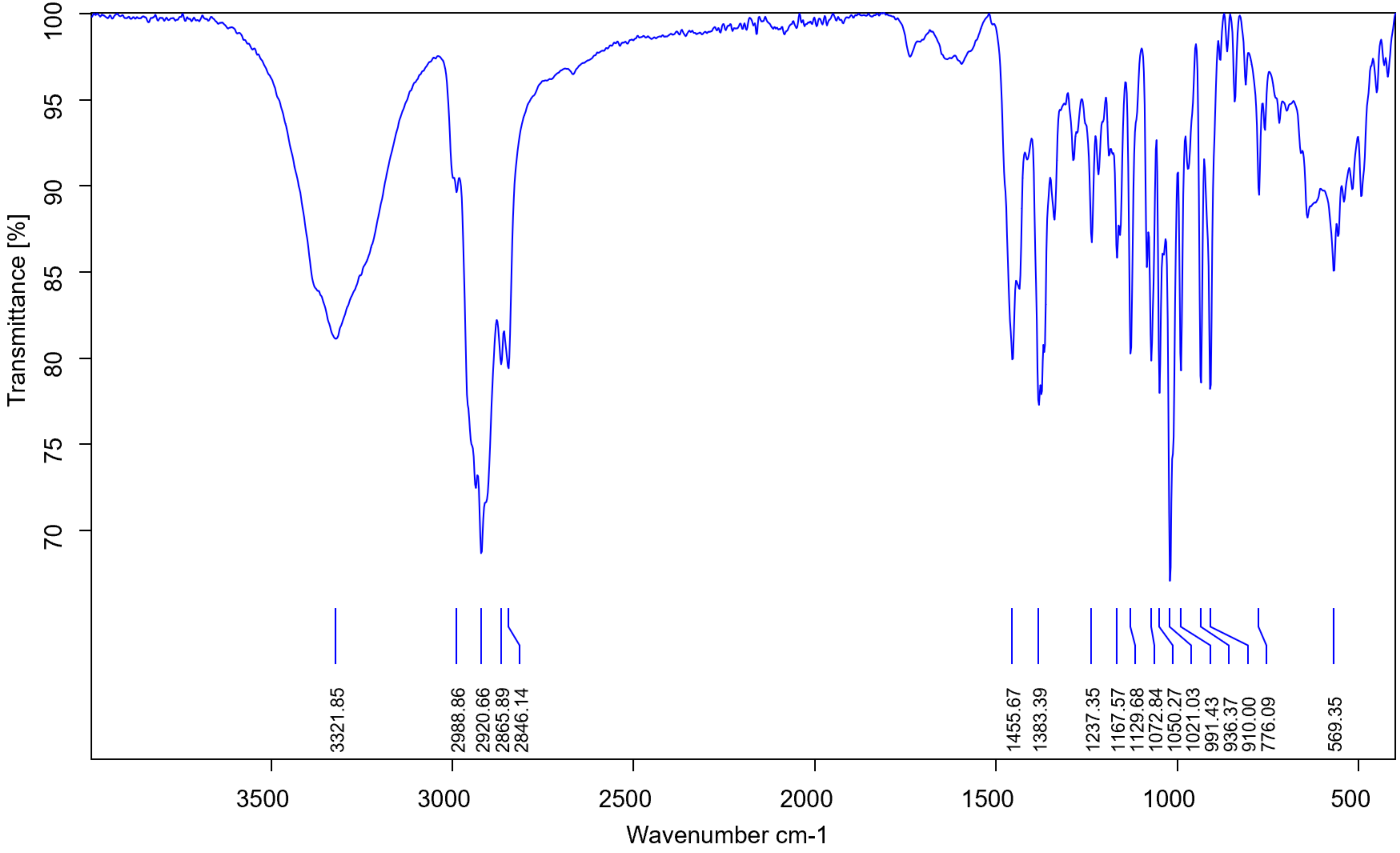

Figure S17. IR (ATR) of **6**.

^{1}H NMR, DEPT 135, 13C NMR, circular dichroism, X-Ray diffraction and IR of 13

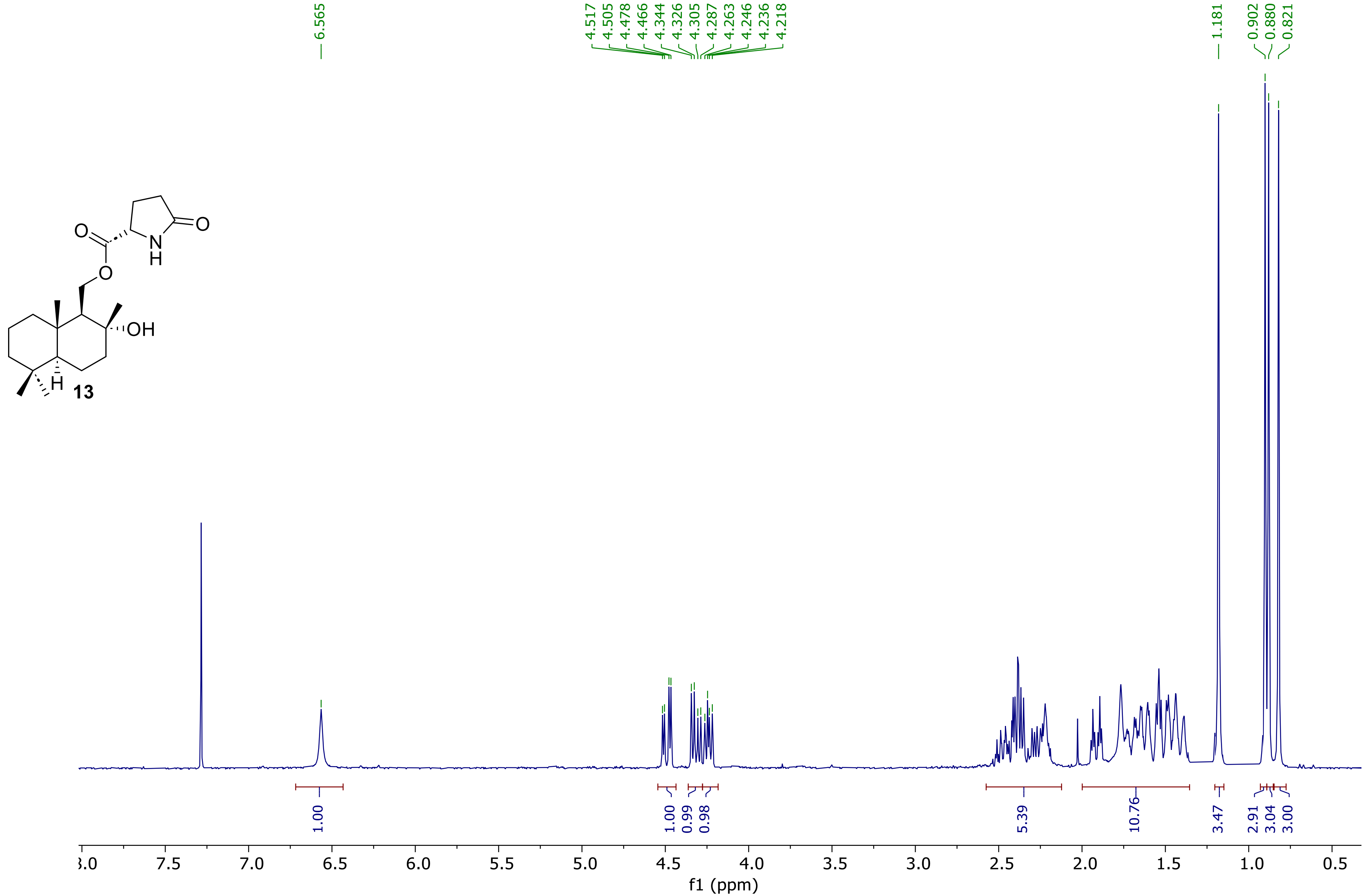

Figure S18. ^{1}H NMR (300 MHz, $CDCl_3$) of **13**.

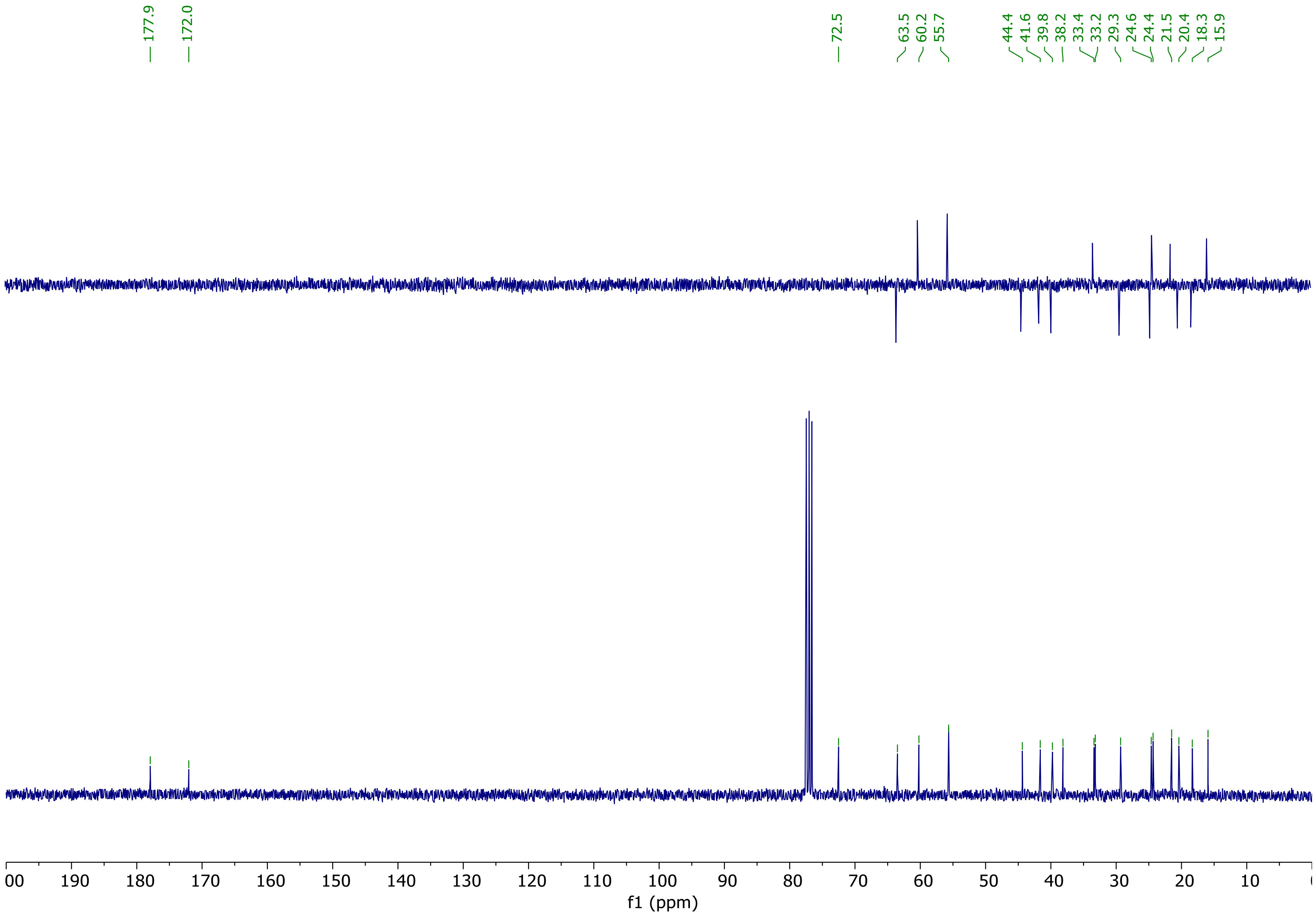

Figure S19. ^{13}C NMR and DEPT (125 MHz, $CDCl_3$) of **13**.

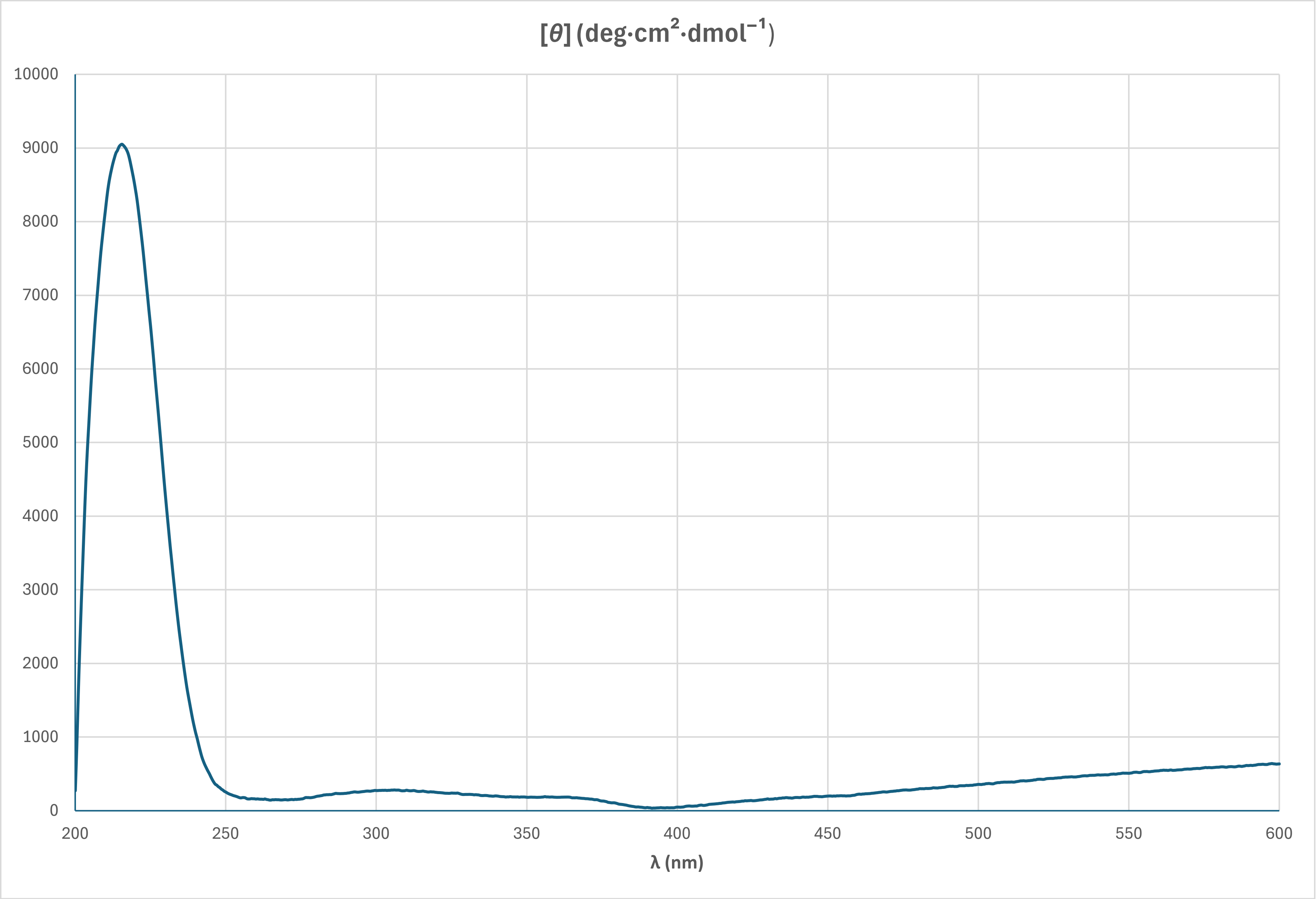

Figure S20. Circular dichroism spectrum (MeOH) of **13**.

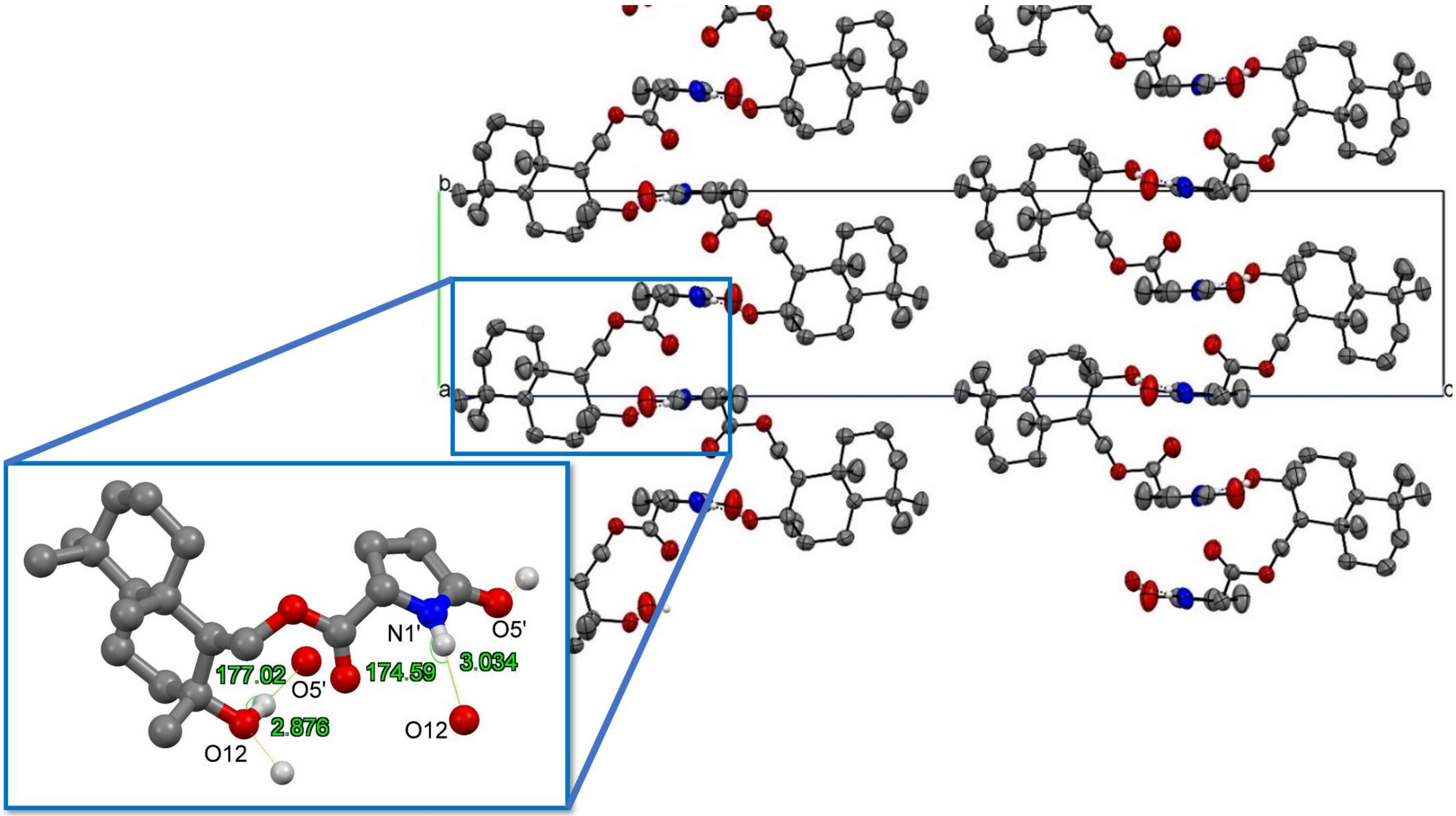

Figure S21. Crystalline Organization of **13**: Polar and Hydrophobic Interactions Between Molecular Planes. **N1'–H1'···O12** (symmetry code: 1–x, –½+y, 3/2–z) with a donor–acceptor distance (D···A) of 3.035(4) Å and a bond angle (∠D–H···A) of 175(4)°, **O12–H12···O5'** (symmetry code: –x, ½+y, 3/2–z) with a D···A distance of 2.876(4) Å and a bond angle of 177(4)°.

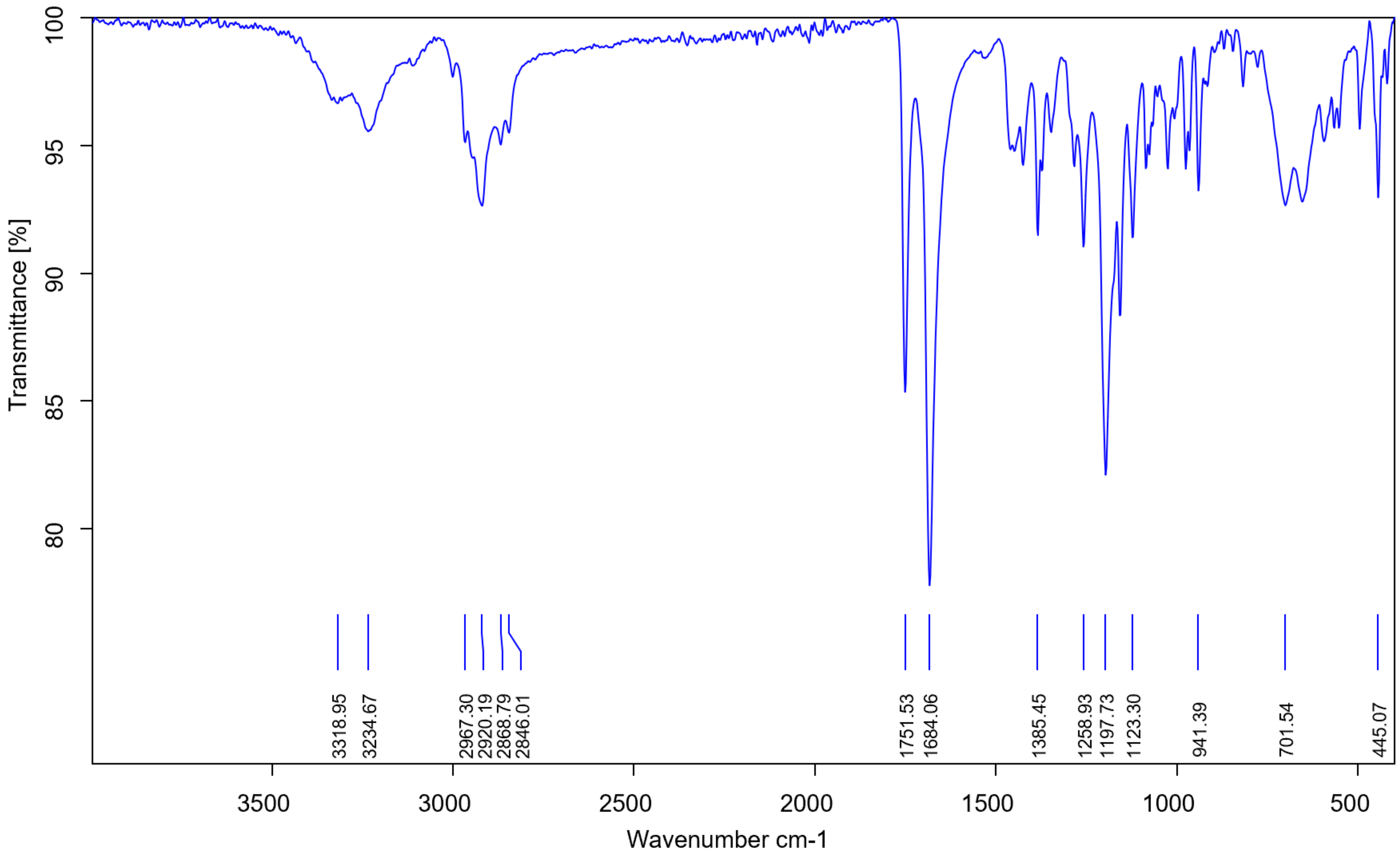

Figure S22. IR (ATR) of **13**.

^{1}H NMR, DEPT 135, ^{13}C NMR, COSY, HMBC, HSQC and NOESY of 14

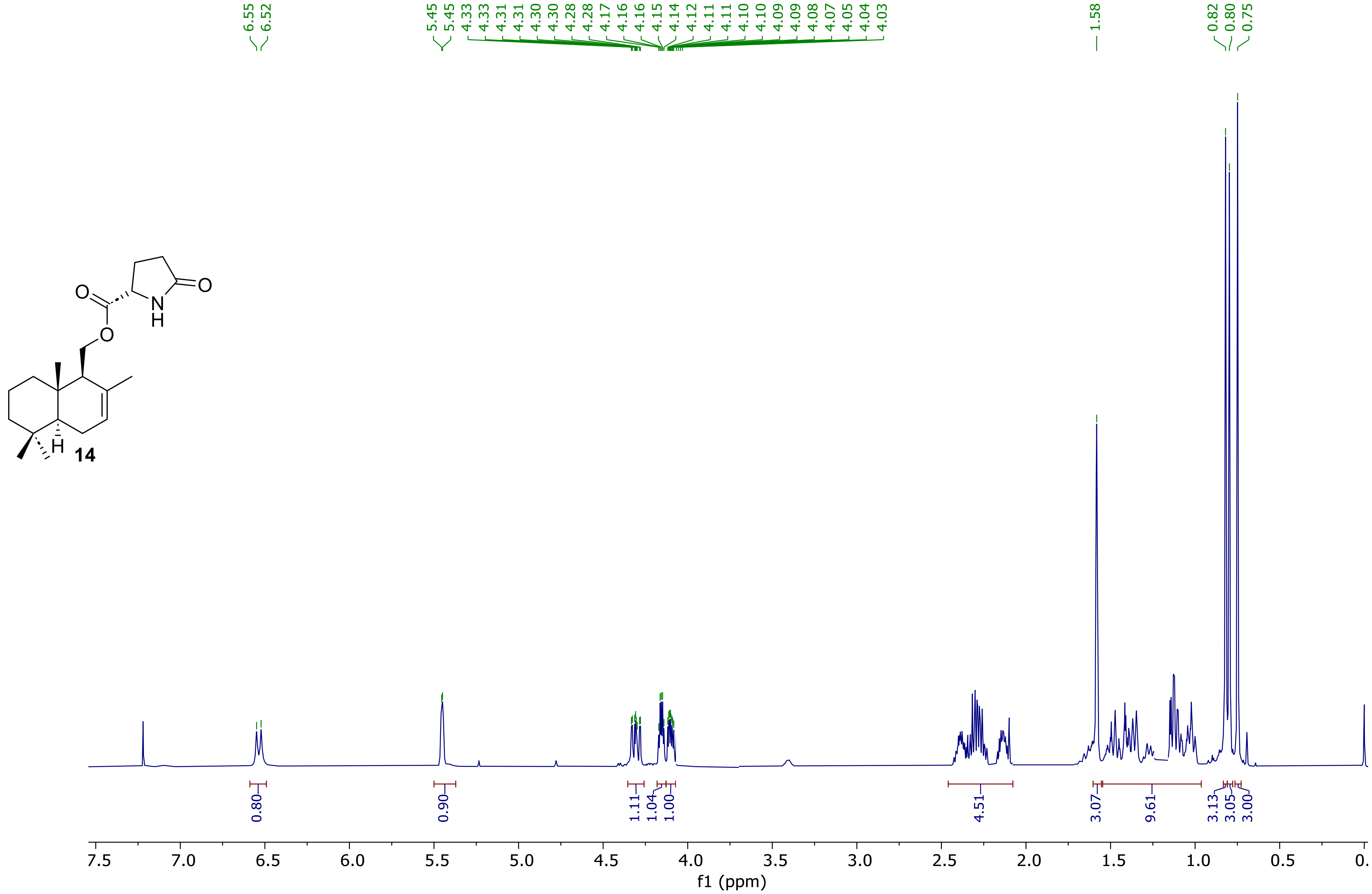

Figure S23. ^{1}H NMR (600 MHz, $CDCl_3$) of **14**.

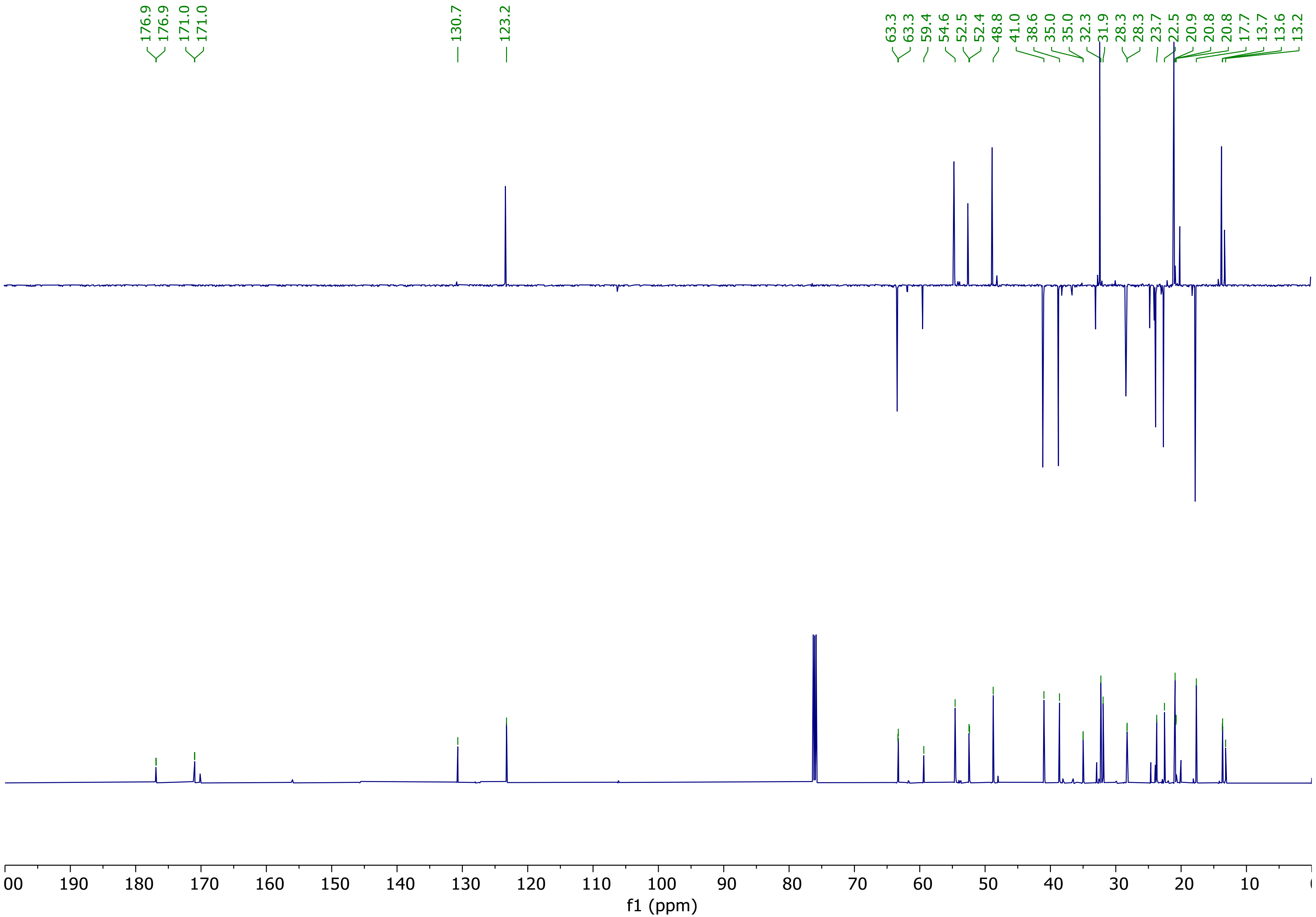

Figure S24. ^{13}C NMR and DEPT (125 MHz, $CDCl_3$) of **14**.

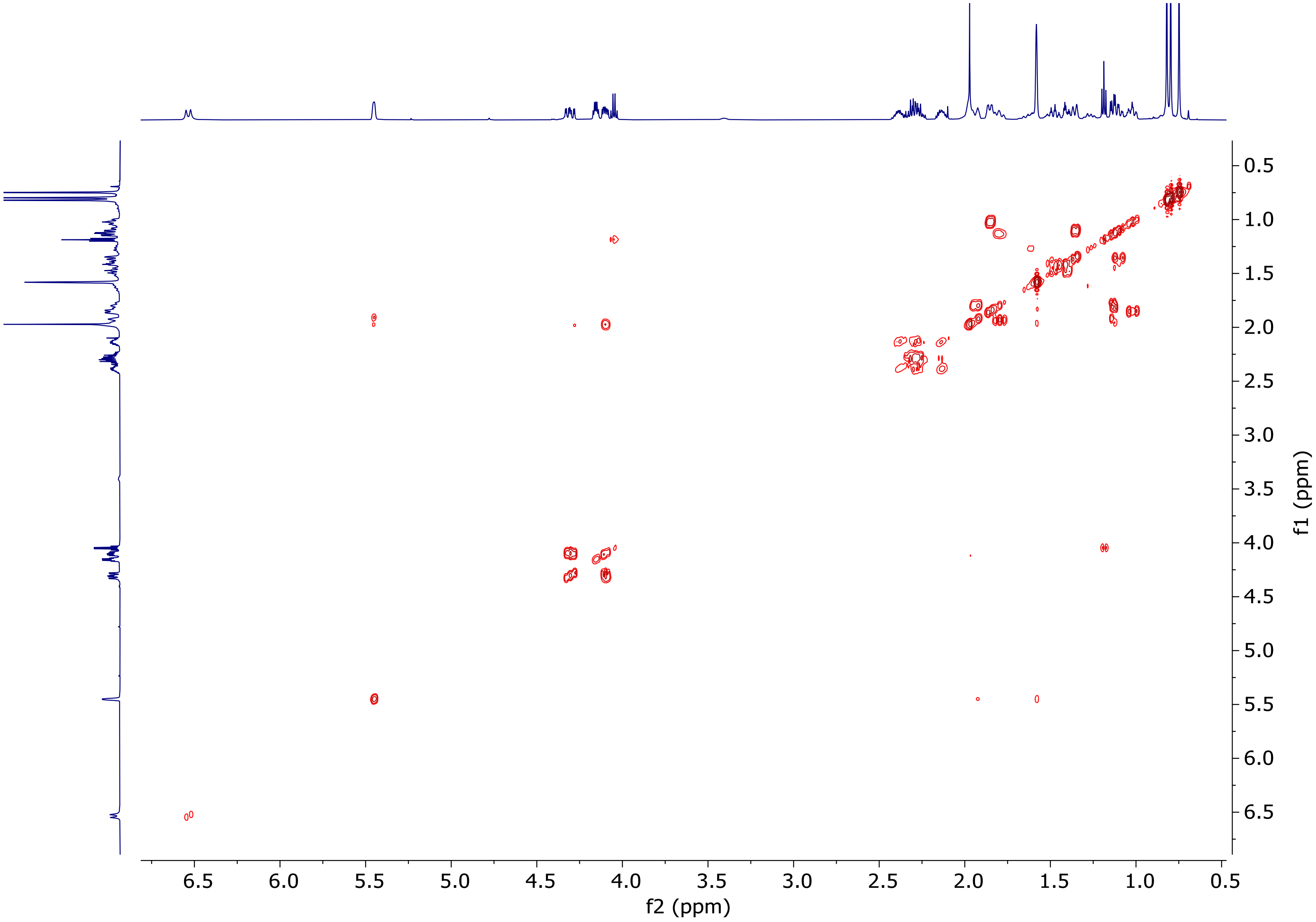

Figure S25. 2D NMR (COSY) (600 MHz, $CDCl_3$) of **14**.

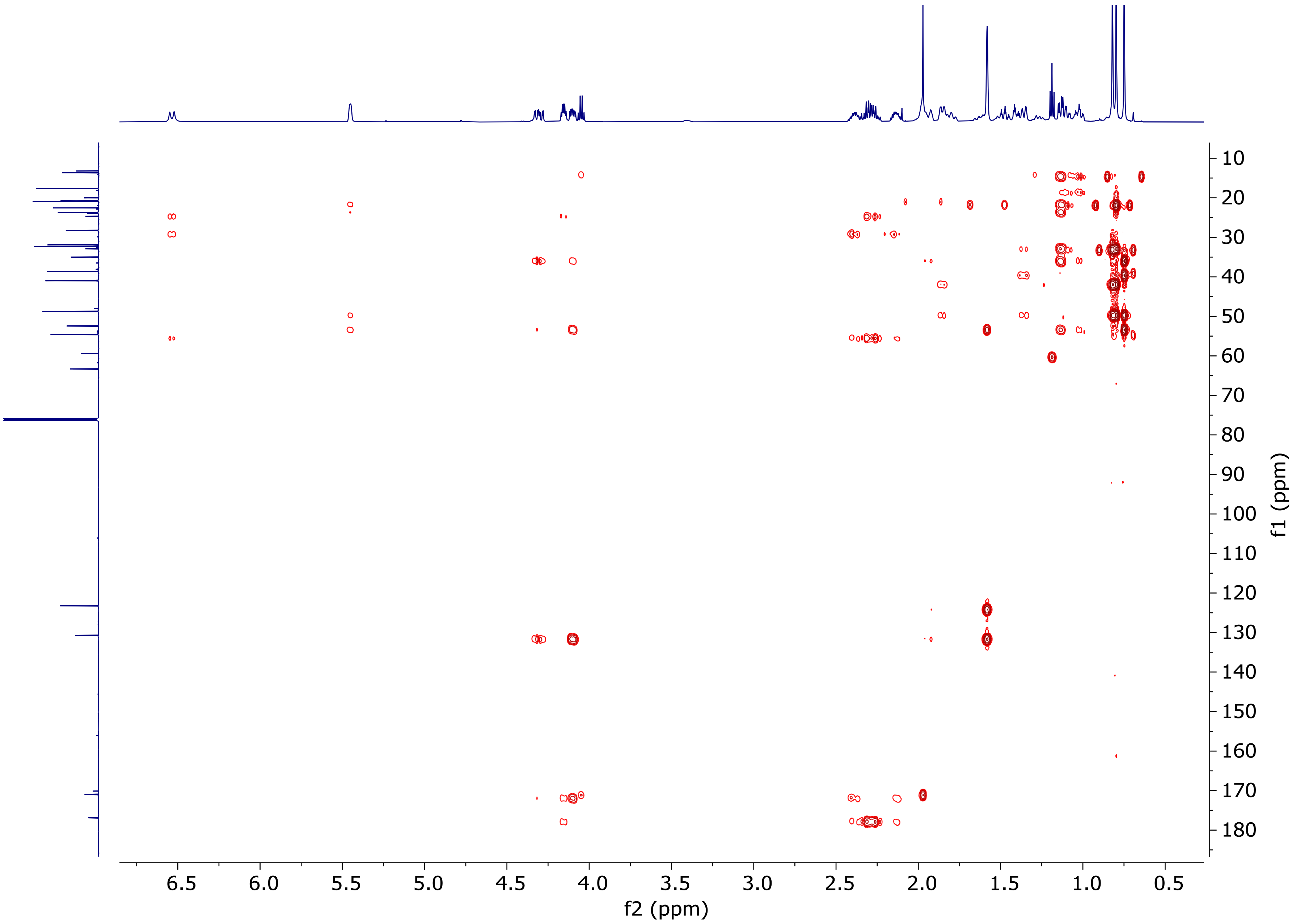

Figure S26. 2D NMR (HMBC) (600 MHz, $CDCl_3$) of **14**.

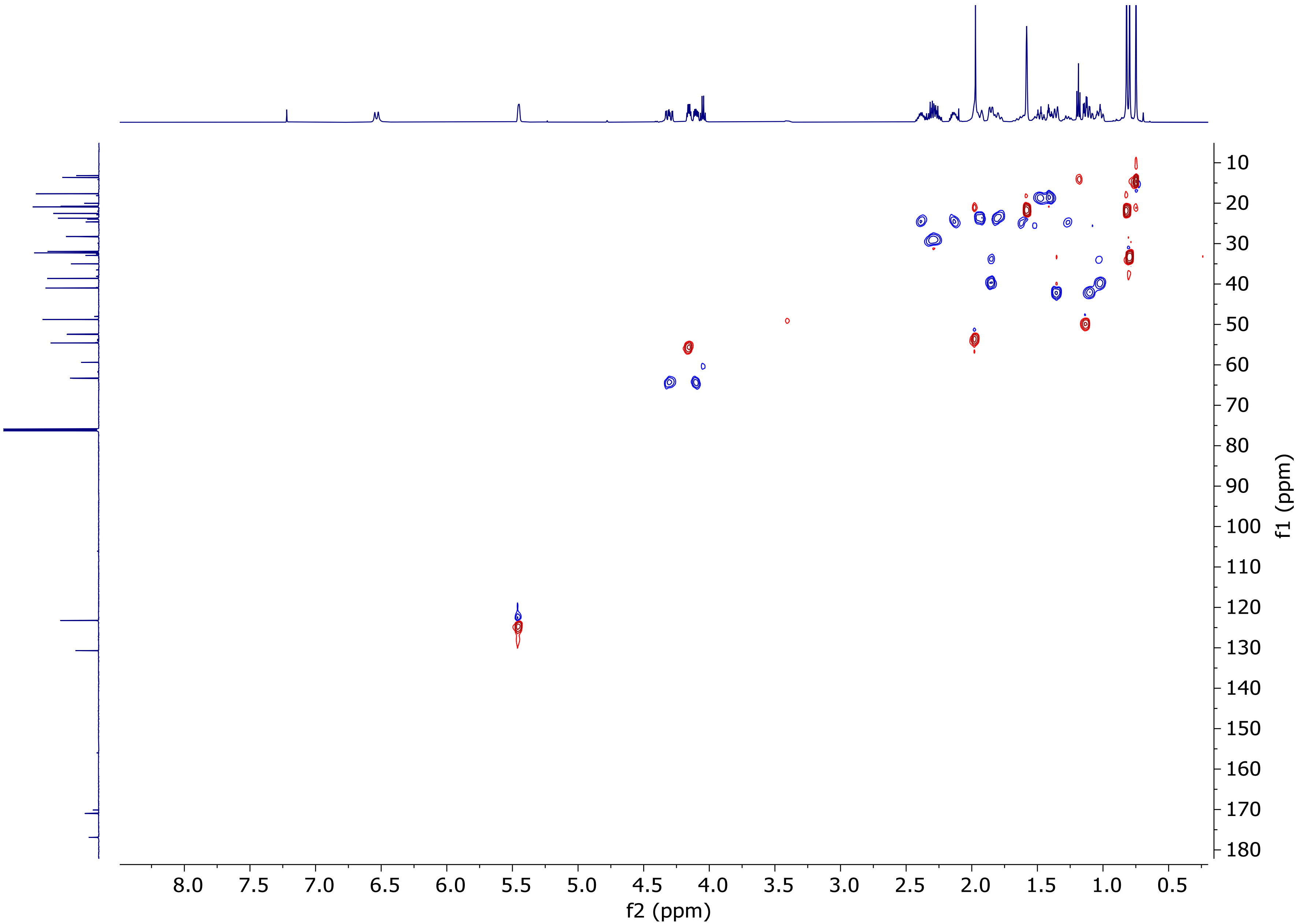

Figure S27. 2D NMR (HSQC) (600 MHz, $CDCl_3$) of **14**.

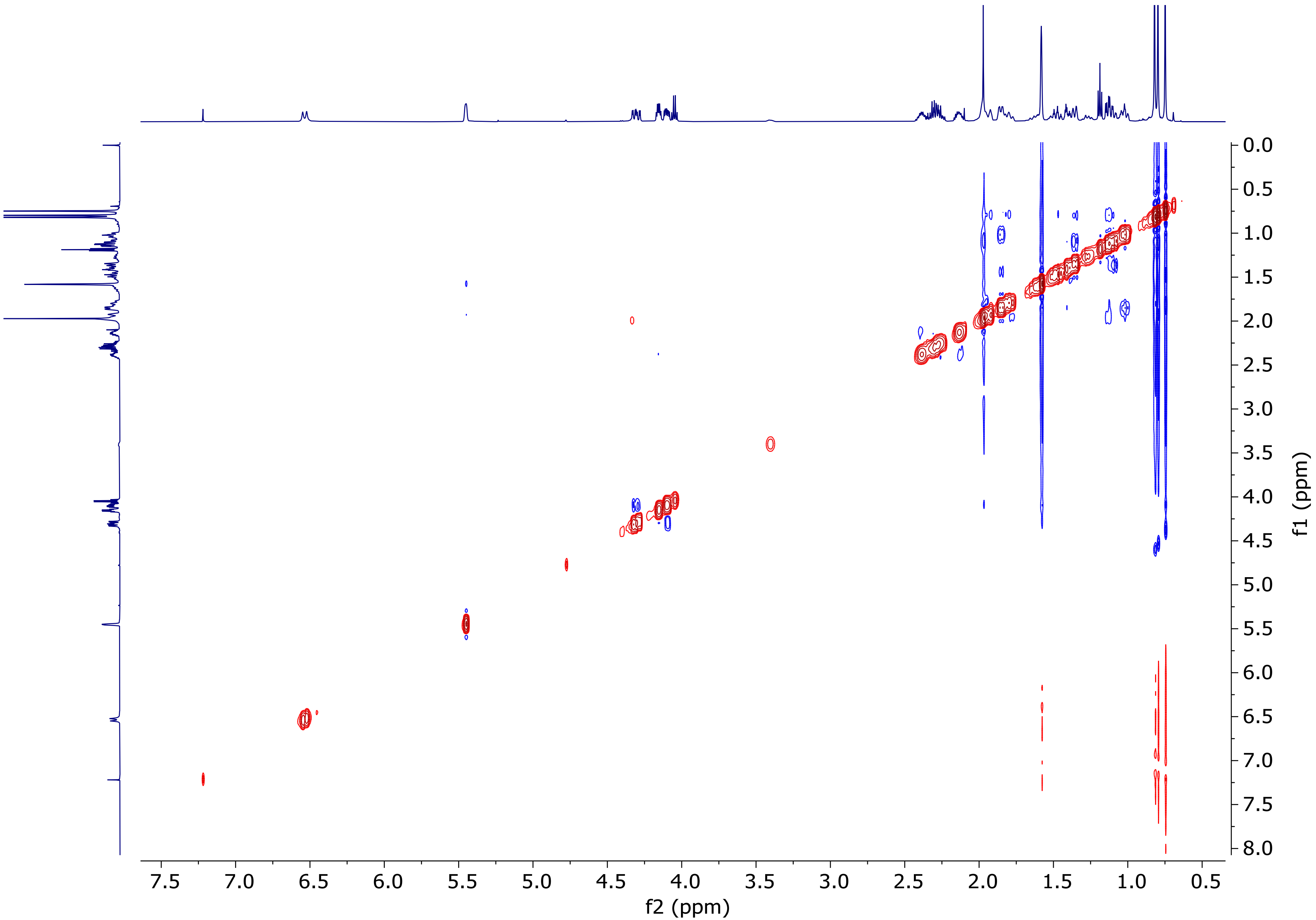

Figure S28. 2D NMR (NOESY) (600 MHz, $CDCl_3$) of **14**.

¹H NMR, DEPT 135, ¹³C NMR, HSQC, COSY and IR of 5

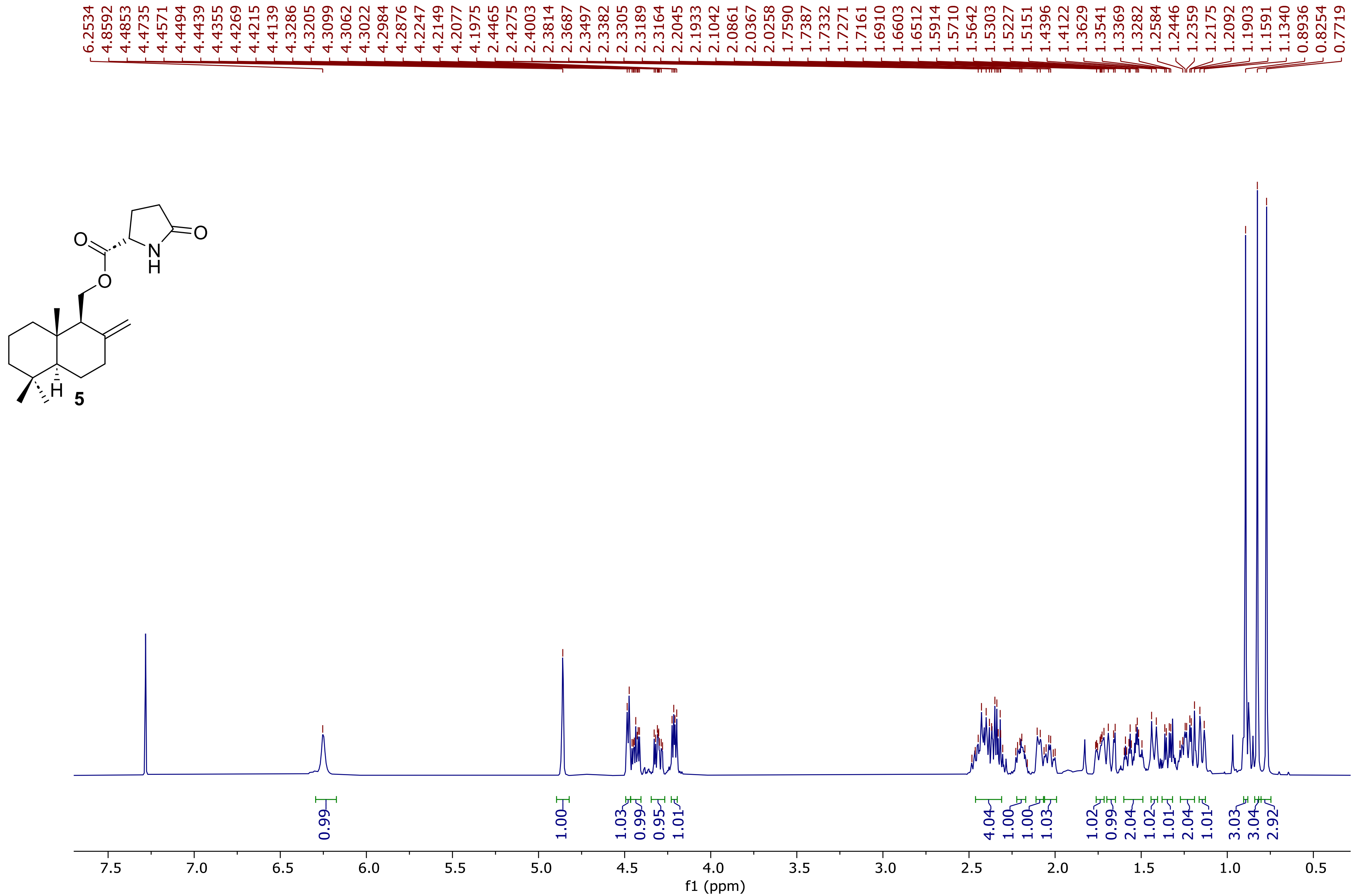

Figure S29. ¹H NMR (500 MHz, $CDCl_3$) of **5.**

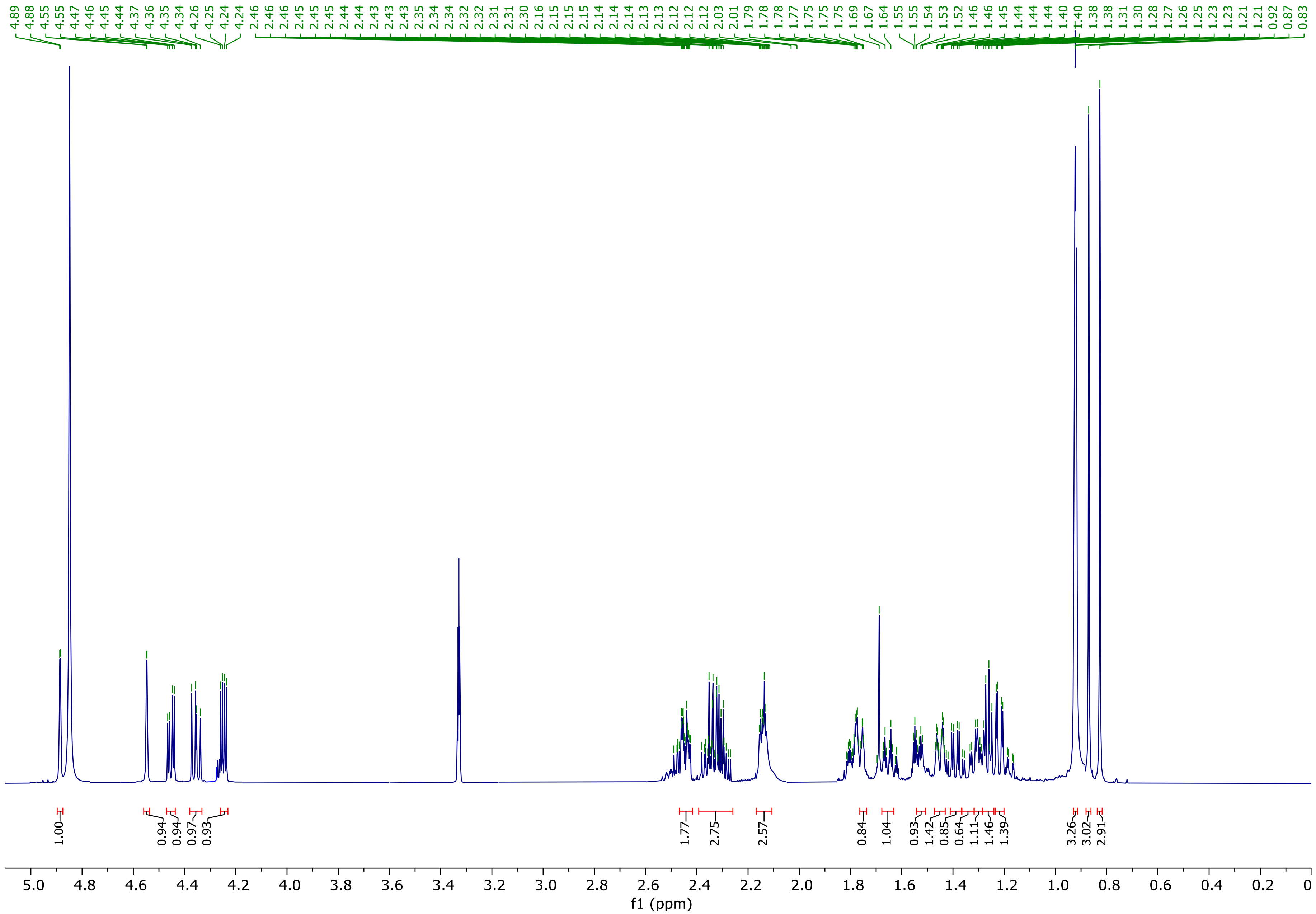

Figure S30. ^{1}H NMR (600 MHz, MeOD-d6) of **5.**

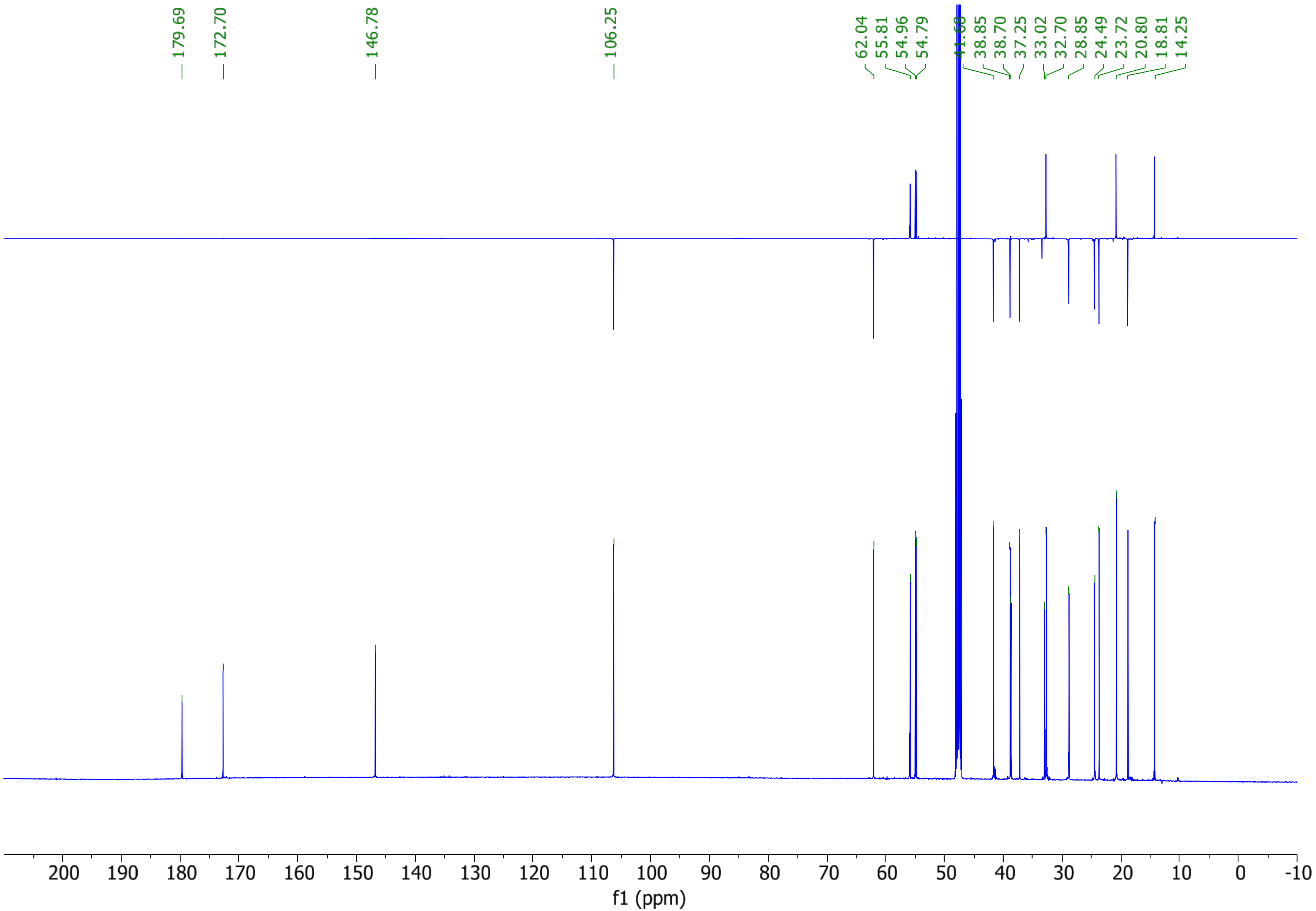

Figure S31. ^{13}C NMR (150 MHz, MeOD-d6) of **5.**

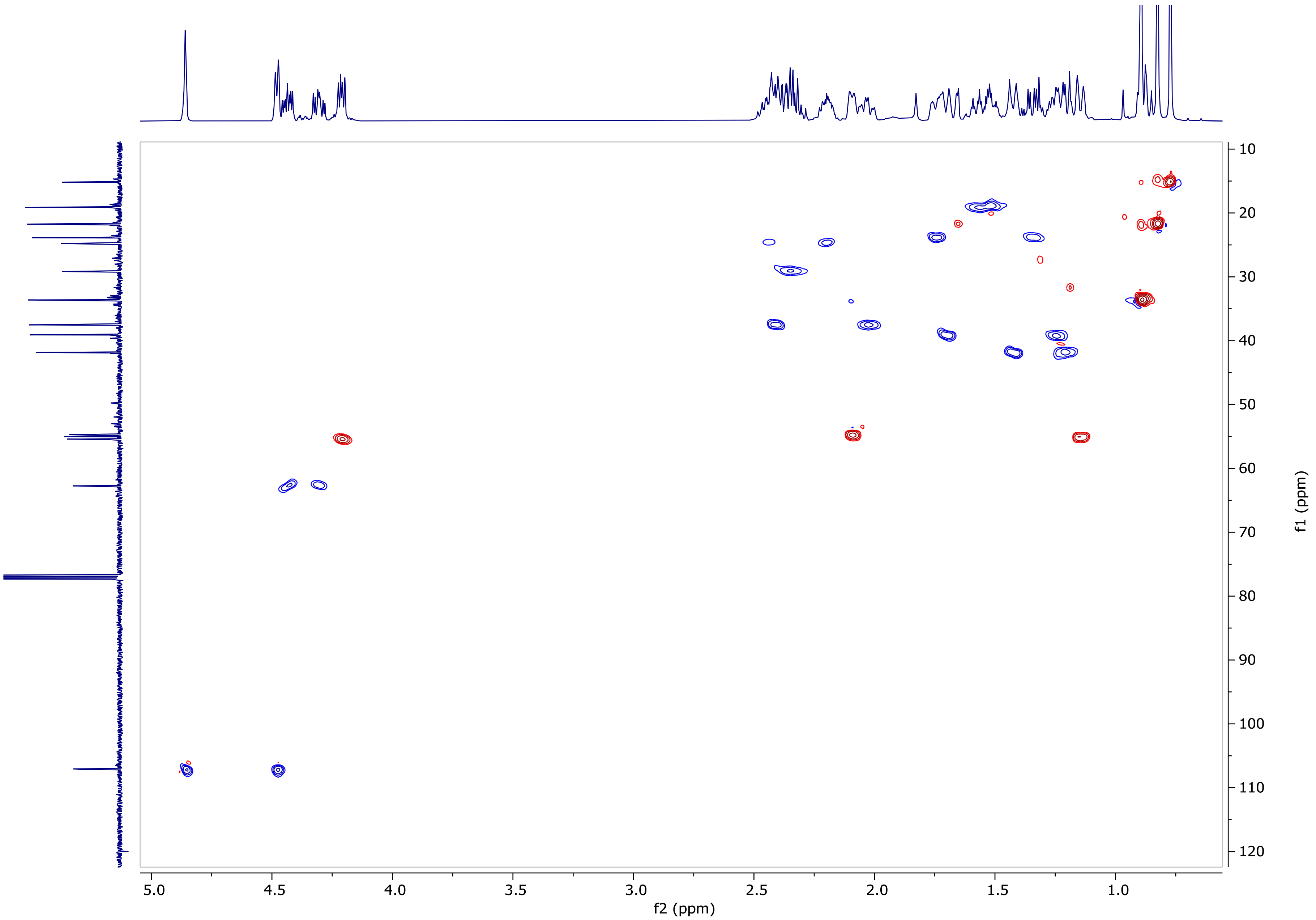

Figure S32. 2D NMR (HSQC) (600 MHz, MeOD-d6) of **5**

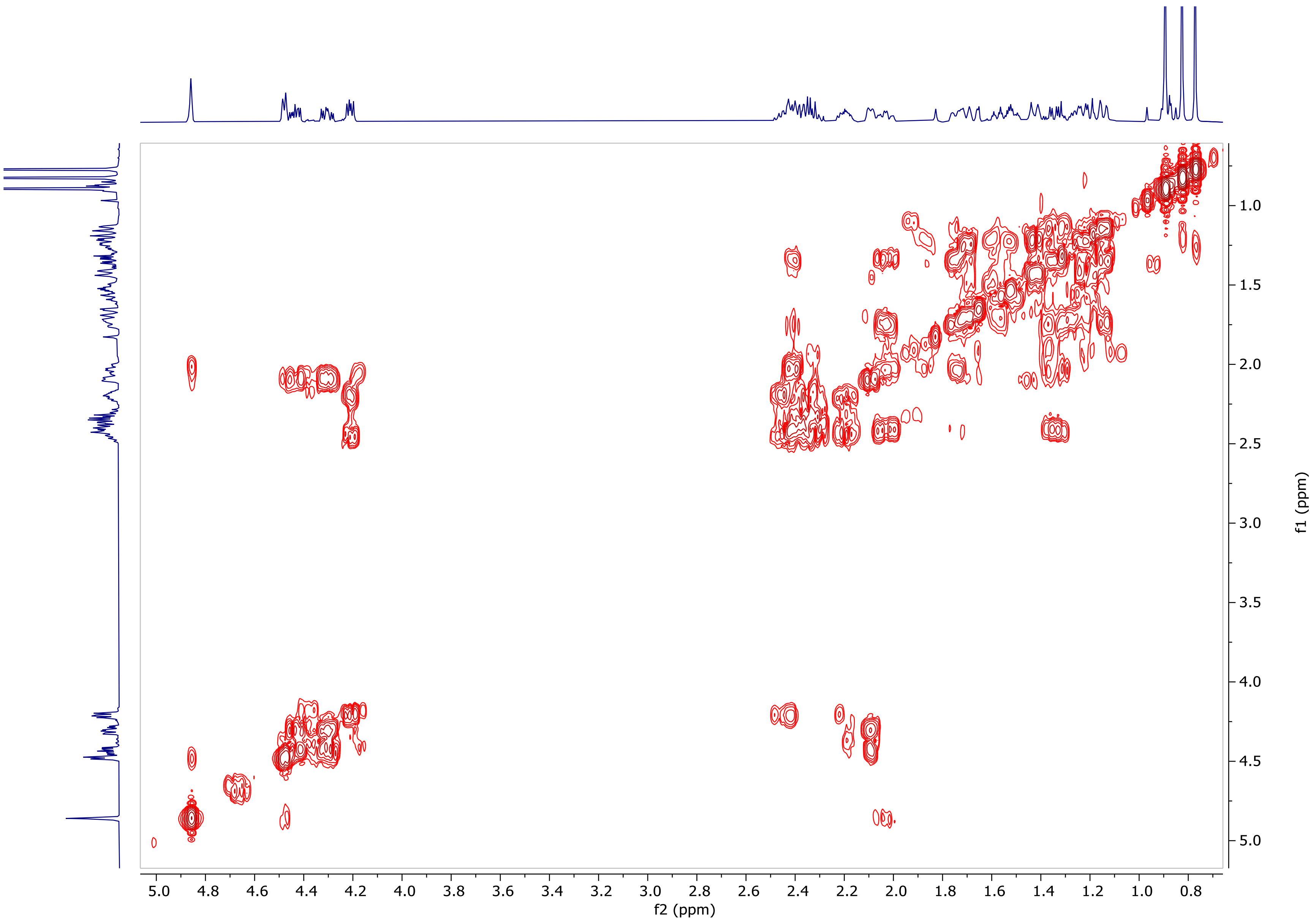

Figure S33. 2D NMR (COSY) (600 MHz, MeOD-d6) of **5**

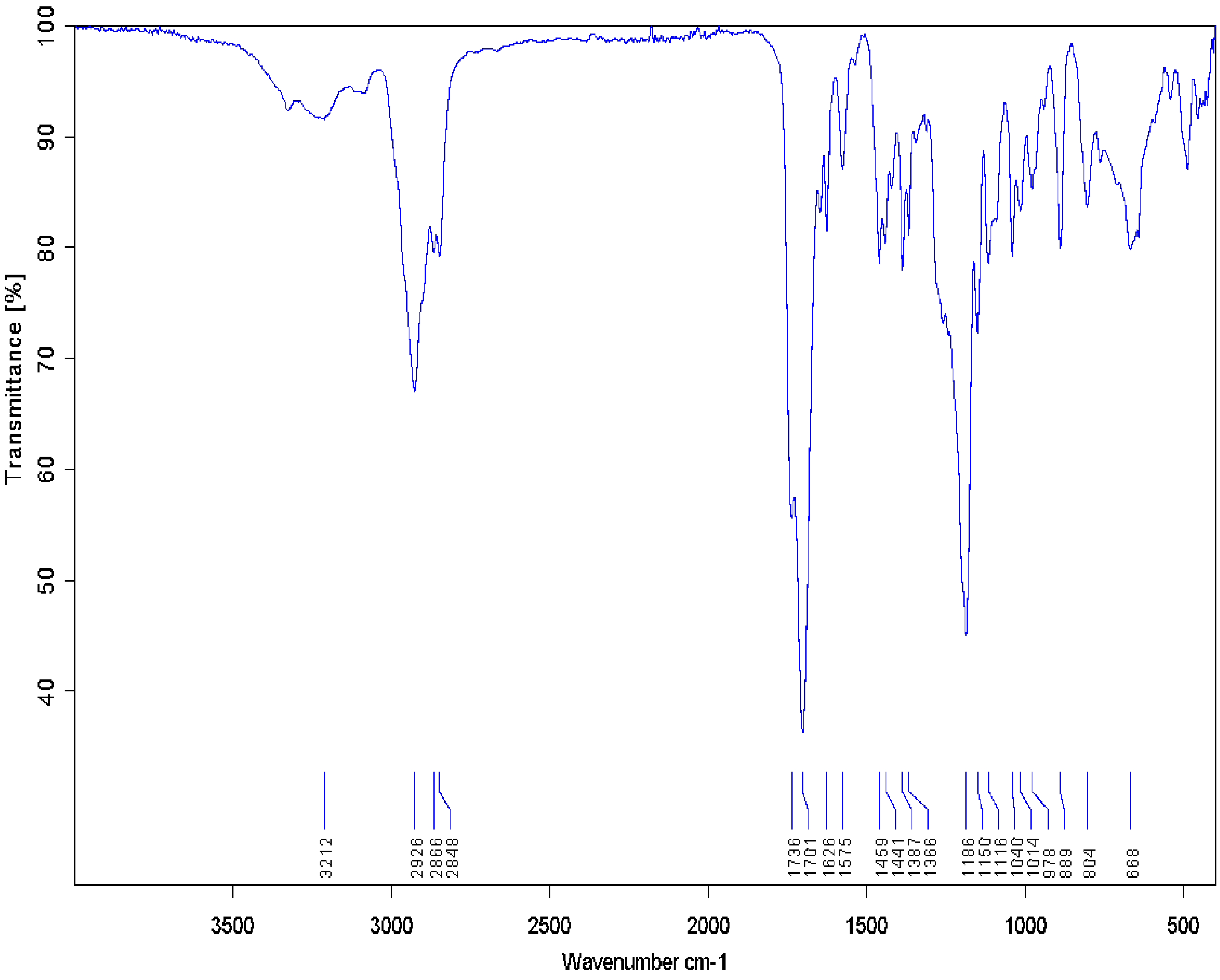

Figure S34. IR (ATR) of **5**

HPLC chromatogram for the separation of 5 and 14.

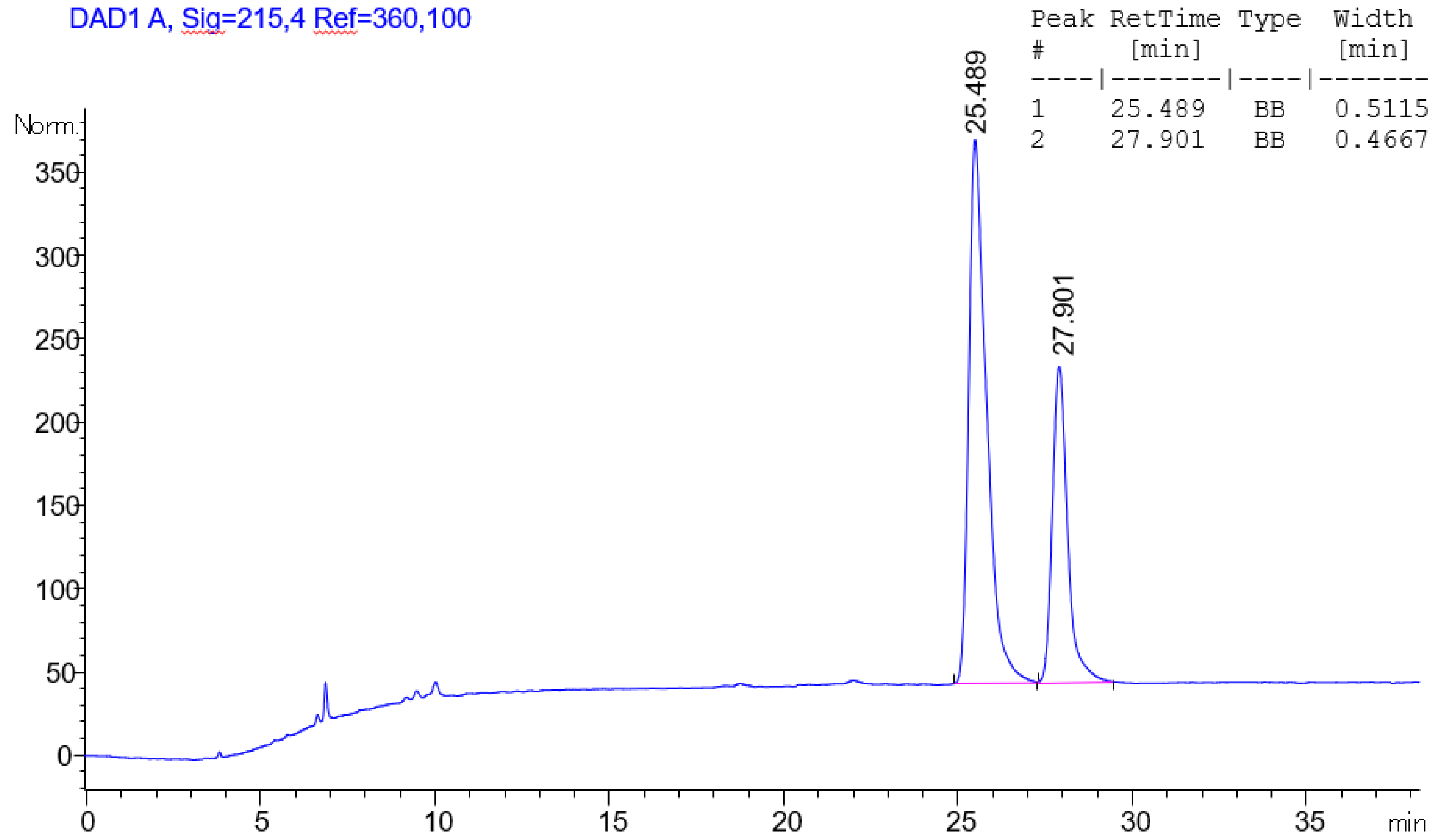

Peak #	RetTime [min]	Type	Width [min]	Area [mAU*s]	Height [mAU]	Area %
1	25.489	BB	0.5115	1.36584e4	327.10498	76.998
2	27.901	BB	0.4667	4140.68018	190.67827	23.0020
				1.77980e4		

Figure S35. HPLC chromatogram for the separation of **5** and **14**.
